# Pharmacophylogenetic insights into *Scutellaria strigillosa* Hemsl.: chloroplast genome and untargeted metabolomics, quantitative analysis and antibacterial analysis

**DOI:** 10.3389/fpls.2024.1472204

**Published:** 2024-09-25

**Authors:** Jie Shen, Panpan Li, Hairong Chu, Yong Li, Xiangying Meng, Zhenpeng Li, Jiayao Dou, Wentao Wang, Chenyang Liu, Peigen Xiao, Chunnian He, Zhengjun Yi

**Affiliations:** ^1^ School of Medical Laboratory, Shandong Second Medical University, Weifang, China; ^2^ Key Laboratory of Bioactive Substances and Resources Utilization of Chinese Herbal Medicine, Ministry of Education, Institute of Medicinal Plant Development, Chinese Academy of Medical Sciences, Peking Union Medical College, Beijing, China; ^3^ Key Laboratory of Immune Microenvironment and Inflammatory Disease Research in Universities of Shandong Province, School of Basic Medical Sciences, Shandong Second Medical University, Weifang, China; ^4^ Experimental Center for Medical Research, Shandong Second Medical University, Weifang, China

**Keywords:** *Scutellaria strigillosa*, chloroplast genome, metabolomic, quantitative analysis, antibacterial activity

## Abstract

*Scutellaria strigillosa* Hemsl., known for its traditional use in Chinese herbal medicine, is valued for heat-clearing and detoxifying, promoting diuresis, reducing swelling, alleviating pain, and preventing miscarriage. Despite its historical use, comprehensive studies on pharmacophylogenetic analysis, including genetic and chemical profiles and the antimicrobial activity of *S. strigillosa* are still lacking. Understanding these aspects is crucial for fully realizing its therapeutic potential and ensuring sustainable use. This study aims to elucidate these aspects through comparative genomics, metabolomics, and antimicrobial assays with *Scutellaria baicalensis* Georgi and *Scutellaria barbata* D. Don. The chloroplast genome of *S. strigillosa* was assembled, measuring 152,533 bp, and revealing a high degree of conservation, especially in the protein-coding regions, and identified four regions *trnK(UUU)*-*rps16*, *trnN(GUU)*-t*rnR(ACG)*, *accD*-*psaI*, *psbE*-*petL*) of variability that could serve as phylogenetic markers. The phylogenetic analysis revealed a closer genetic relationship of *S. strigillosa* with *S. tuberifera* and *S. scordifolia* than traditionally classified, suggesting a need for taxonomic reevaluation within the genus. UPLC-Q-TOF-MS analysis in negative ion mode was used to explore the chemical diversity among these species, revealing distinct variations in their chemical compositions. *S. strigillosa* shared a closer chemical profile with *S. barbata*, aligning with phylogenetic findings. Metabolomic identification through Progenesis QI software resulted in the tentative identification of 112 metabolites, including a substantial number of flavonoids, diterpenoids, iridoid glycosides, phenylethanoid glycosides, and others. HPLC analysis further detailed the concentrations of 12 actives across the species, highlighting the variation in compound content. *S. strigillosa* shows antibacterial activity against *Staphylococcus aureus* and *Pseudomonas aeruginosa*, similar to *S. baicalensis* root extracts. This research enhances the understanding of the phylogenetic and phytochemical profiles and the antibacterial activity of *S. strigillosa*, offering new insights into its medicinal properties. The findings suggest a need for taxonomic reevaluation within the genus and underscore the potential antibacterial activity of *S. strigillosa* for therapeutic applications. Further studies are encouraged to explore its full medicinal potential and contribute to the sustainable development of *Scutellaria* species.

## 1 Introduction

**Figure 1:**
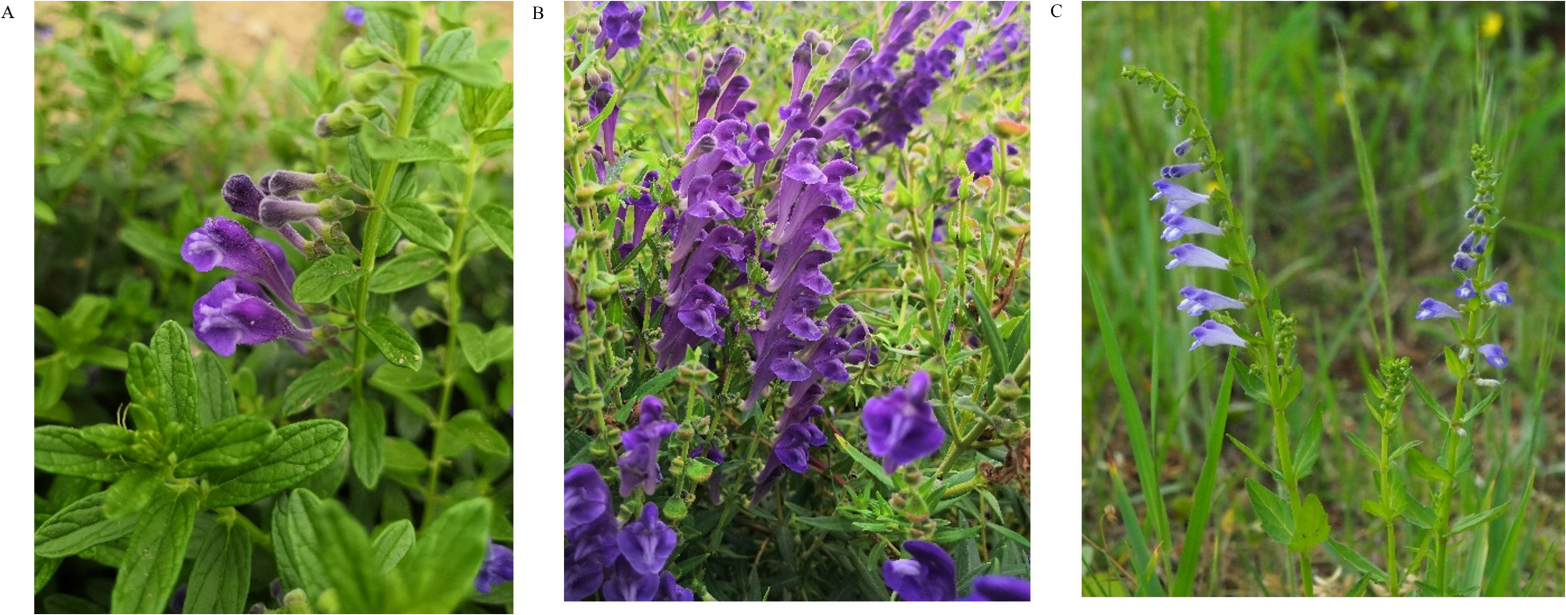
*Scutellaria strigillosa* Hemsl.**(A)**, *Scutellaria baicalensis* Georgi **(B)** and *Scutellaria barbata* (D) Don **(C)**.

*Scutellaria strigillosa* Hemsl. (**Figure 1**) is a perennial herb that thrives on sandy beaches, primarily found in Liaoning, Hebei, Shandong, Jiangsu, Zhejiang, and other regions in China, as well as in Russia, North Korea, and Japan. The whole plant of *S. strigillosa* is used medicinally in folk medicine for heat-clearing and detoxifying, promoting diuresis, reducing swelling, alleviating pain, and preventing miscarriage (Dai et al., 2016; Shen et al., 2021). Modern studies have demonstrated its antibacterial (Zhu et al., 2016), antitumor (Wang et al., 2023), and cardiovascular protective activities *in vitro* and *in vivo* (Li et al., 2019). Besides its medicinal value, *S. strigillosa* is resistant to harsh environments, including wind, drought, salt, and alkali, making it ideal for growth in barren sandy soils by the sea. It also adapts well to salt stress and is effective in desalination (Song et al., 2019). Its dark green leaves and long flowering period (May to October) add ornamental value, making it a popular choice in coastal urban landscaping. Despite numerous pharmacological studies, the genetic, chemical composition, and antibacterial activity of *S. strigillosa* remains insufficiently explored, limiting a comprehensive understanding of its medicinal potential.

According to pharmacophylogenetic theory, phylogenetically related plants often share similar therapeutic effects and chemical compositions. *S. strigillosa* belongs to the genus *Scutellaria*, renowned for its significant medicinal value. Notably, *Scutellaria baicalensis* Georgi and *Scutellaria barbata* D. Don, widely studied species within this genus, are officially recorded in successive editions of the Chinese Pharmacopoeia. *S. baicalensis* is acclaimed for its neuroprotective, hepatoprotective, antitumor, antibacterial, antiviral, and antioxidant effects (Shen et al., 2021). *S. barbata*, found in Southern China, is traditionally used for its anticancer, bacteriostatic, antiviral, anti-inflammatory, antioxidant, and immune-enhancing activities (Shen et al., 2021). Although *S. strigillosa* is closely related *to S. baicalensis* and *S. barbata*, these species exhibit distinct phytochemical profiles that contribute to their therapeutic potential. In addition, *S. strigillosa*, despite its long history in traditional medicine, remains less explored in scientific research. This study aims to elevate the understanding of *S. strigillosa* by positioning it within a comparative genomic, metabolomic, and antibacterial activity framework alongside its more studied species in the *Scutellaria* genus, *S. baicalensis*, and *S. barbata* D. Don.

The genus *Scutellaria* exhibits rich genetic diversity, but the phylogenetic relationships within the genus, including the systematic position of *S. strigillosa*, remain unclear (Salimov et al., 2021; Shen et al., 2022; Yang et al., 2022). The chloroplast genome, a key resource in plant genetic research, provides insights into these relationships and evolutionary patterns, especially within *Scutellaria*. This study explores the chloroplast genome of *S. strigillosa* to clarify its phylogenetic placement and its evolutionary significance. Furthermore, metabolomics, which involves the comprehensive profiling of metabolites in a biological system, stands at the forefront of linking genotype to phenotype (Xu et al., 2024). Metabolomic analysis using advanced techniques like LC-MS and HPLC was conducted to profile secondary metabolites, which are crucial for the medicinal properties of *Scutellaria*. Modern research shows that heat-clearing and detoxifying drugs are effective in treating febrile conditions and are commonly used for bacterial infections, suggesting that medicinal plants with these properties may have strong antibacterial effects (Heng et al., 2023). Most *Scutellaria* species are known for their heat-clearing and detoxifying effects (Shen et al., 2021), with *S. baicalensis* and *S. barbata* proven effective against various pathogens (Millar et al., 2021; Shen et al., 2021). *S. strigillosa* is also believed to have similar properties. Although its antibacterial effects are not yet confirmed, this study aims to evaluate the antibacterial activities of *S. strigillosa*, *S. baicalensis*, and *S. barbata* against *Staphylococcus aureus* and *Pseudomonas aeruginosa*, focusing on the potential of *S. strigillosa*.

This study enhances the understanding of the medicinal value of *S. strigillosa* by positioning it within a comparative genomic, metabolomic, and antibacterial framework alongside *S. baicalensis* and *S. barbata*. The complete chloroplast genome of *S. strigillosa* was sequenced for the first time using Illumina technology, and comparative analyses explored the unique characteristics of the chloroplast genomes of *S. strigillosa*, *S. barbata*, and *S. baicalensis*. A phylogenetic tree was constructed to elucidate the phylogenetic position of *S. strigillosa* within the genus *Scutellaria*. UPLC-Q-TOF-MS was employed to determine the metabolites present in these species, while HPLC analysis quantified 12 main flavonoids. Finally, the antibacterial activities of these species against *Staphylococcus aureus* and *Pseudomonas aeruginosa* were evaluated, expanding our understanding of the genus *Scutellaria* and promoting more in-depth research on *S. strigillosa*.

## 2 Materials and methods

### 2.1 Plant materials

The fresh whole plants of *S. strigillosa* were collected in Yangkou Beach, Qingdao City, Shandong Province, China. The fresh whole plants of *S. bancalensis* were collected in Baihua Mountain, Beijing, China. The entire plants of *S. barbata* were collected from the Beijing Medicinal Botanical Garden, Beijing City, China. The collection of wild-grown Scutellaria plants was performed with the permission of landowners. The samples were authenticated by Professor Chunian He (Institute of Medicinal Plant Development, Chinese Academy of Medical Sciences, Peking Union Medical College) and deposited in the pharmacophylogeny research center (Institute of Medicinal Plant Development, Chinese Academy of Medical Science, Peking Union Medical College, Beijing, China).

### 2.2 Sample collection, DNA extraction, and sequencing

The fresh leaves of *S. strigillosa* were harvested for genomic DNA extraction. The samples were sourced from Weihai City in Shandong Province, China. Immediately after collection, these leaves were desiccated using silica gel to preserve their integrity for DNA extraction. The extraction process was carried out utilizing the Plant Genomic DNA Kit (Huayueyang, Beijing, China), adhering strictly to the provided protocol. To evaluate the DNA quality, we conducted 1% (w/v) agarose gel electrophoresis. DNA concentrations were accurately measured using a NanoPhotometer^®^ spectrophotometer (IMPLEN, CA, USA) and a 2.0 Fluorometer (Life Technologies, CA, USA). Upon confirming the high quality of the DNA, we proceeded to construct a library and performed sequencing on an Illumina platform, yielding 150 bp paired-end reads.

### 2.3 Chloroplast genome assembly, annotation and structure analysis

K-values of 21, 55, 85, and 115 were employed in the GetOrganelle software to selectively obtain the desired outcomes (Jin et al., 2018). For the chloroplast genome assembly, the complete genome of *Scutellaria baicalensis* Georgi (NC_027262.1), sourced from the National Center for Biotechnology Information (NCBI), served as the reference.

The chloroplast annotation of the assembled sequences was conducted using GeSeq (Michael et al., 2017), accessible at (https://chlorobox.mpimp-golm.mpg.de/geseq.html). In this process, the BLAT threshold was set to a matching identity of over 85% for protein-coding genes. Similarly, for rRNA/tRNA genes, the BLAT threshold was also established at a matching identity greater than 85%. Beyond the default known database in GeSeq, the annotation also incorporated the complete genome of *S. baicalensis* from the NCBI database. Following the annotation results from GeSeq, OGDRAW (available at [http://ogdraw.mpimp-golm.mpg.de/]) was utilized to generate a circular physical map of the chloroplast genome (Stephan et al., 2019). The GC content of the chloroplast genome was analyzed using the MEGA-X software (Kumar et al., 2018).

### 2.4 Interspecific comparison and phylogenetic analysis

The complete chloroplast genomes of *S. baicalensis* (MF521633), *S. barbata* (MW376479) were downloaded from the NCBI database. IRscope (https://irscope.shinyapps.io/irapp/) was used to visualize the gene differences at the boundaries of the junction sites of the 3 chloroplast genomes (Amiryousefi et al., 2018). The mVISTA web interface (http://genome.lbl.gov/vista/mvista/submit.shtml) was used to align and compare the three cp genome:*S. strigillosa*, *S. baicalensis* (MF521633), *S. barbata* (MW376479) sequences (Mayor et al., 2000). Shuffle-LAGAN was selected as the alignment program to detect sequence rearrangements and inversions. The divergence among different chloroplast genomes and identifying mutational hotspots were performed by quantifying nucleotide variability in DnaSP v6.12.03 (Rozas et al., 2017). The window length was 600 bp, with a 200 bp step size.

In this study, in addition to the newly sequenced chloroplast genomes of *S. strigillos*, 27 chloroplast genomes were downloaded from NCBI to construct a chloroplast phylogenetic tree. The sequences were aligned using MAFFT (v7.471) (https://mafft.cbrc.jp/alignment/server/index.html) with the default settings. Maximum likelihood (ML) phylogenetic inference was made using IQ-TREE software (Nguyen et al., 2015) with 1000 bootstrap replicates based on the TVM+F+I+R4 nucleotide substitution model to assess branch support. The MrModeltest 2.3 (Nylander, 2004) calculates the best-fit model based on Akaike’s information criterion. The Bayesian inference (BI) phylogenetic trees were analyzed using Phylosuite (Zhang et al., 2020). The analysis was conducted with MrBayes v 3.2.5, adopting the GTR+F+I+G4 model. The BI analysis followed these parameters: The Markov chain Monte Carlo algorithm ran for 1,000,000 generations, with sampling conducted every 1,000 generations. The initial 25% of the samples, classified as burn-in samples, were discarded. The remaining samples were then utilized to build the majority-rule consensus tree and to calculate the posterior probabilities for each branch.

### 2.5 UPLC-Q-TOF-MS analysis

The entire plant of *S. strigillosa, S. baicalensis*, and *S. barbata* was processed for analysis. In addition, *S. baicalensis* is relatively large, and its root is the commonly used medicinal part. Previous studies have shown significant differences in the chemical composition between the aerial and root parts of *S. baicalensis*. To accurately capture these variations, we analyzed these parts separately. In contrast, *S. strigillosa* and *S. barbata* are smaller plants that are commonly used in their entirety, as their roots are underdeveloped. Therefore, we analyzed the whole plants for these two species. The dried samples from these plants were ground to a fine consistency and passed through a 40-mesh sieve. Weighing 25 mg each, these samples were immersed in 25 ml of methanol and subjected to ultrasonication in a water bath for 30 minutes. The samples were allowed to cool to room temperature, and their volume was adjusted as needed. The resulting extracts were then filtered using a 0.25-μm membrane filter. For each subsequent analysis, 1 mL of this prepared extract was utilized. To ensure the reliability of the results, three biological replicates were prepared, each using different individual plants of the same species.

The UPLC-Q-TOF-MS analysis of *S. strigillosa, S. baicalensis*, and *S. barbata* was performed using a state-of-the-art Waters Acquity Ultra High-Performance Liquid Chromatography (UPLC) system, integrated with a Waters Xevo G2-XS Time-of-Flight (TOF) mass spectrometer. Both these instruments were equipped with an electrospray ionization interface, enhancing their analytical capabilities. Chromatographic separation was efficiently achieved using a Waters Acquity BEH C18 column (2.1 mm × 100 mm, 1.7 µm). The mobile phase for the chromatography consisted of two components: 0.1% formic acid in water (designated as solvent A) and methanol (solvent B). The temperature of the column was precisely controlled and maintained at 35°C throughout the analysis. The elution gradient was meticulously programmed as follows: from 0-3 minutes, the concentration of solvent B was increased from 10%-35%; between 3-9 minutes, it was further increased from 35%-70%; from 9-12 minutes, the concentration of solvent B reached from 70%-100%; and finally, from 12-14 minutes, the concentration was held steady at 100% B. The flow rate was set at a constant 0.3 mL/min. Additionally, a photodiode array detector was utilized, scanning across a broad wavelength range of 200 to 400 nm, allowing for detailed detection and analysis of various metabolites in the samples.

The negative mode was employed for the ionization process in the UPLC-Q-TOF-MS analysis, allowing for the scanning of mass spectra in the range of 1,000 to 1,200 m/z. This mode is particularly effective for detecting and analyzing negatively charged ions in the samples. The cone voltage was set to 40 V, and the capillary voltage was calibrated at 2.4 kV, ensuring optimal ionization conditions. The temperatures of the source and the desolvation unit were carefully controlled, with the source temperature set at 100°C and the desolvation temperature at a higher 350°C. This temperature setting plays a crucial role in ionization efficiency and the subsequent transportation of ions into the mass spectrometer. The flow rates of the desolvation and cone gases were also finely tuned, with the desolvation gas flowing at 900 L/h and the cone gas at 50 L/h, optimizing the removal of solvent vapors and enhancing the ionization process. Leucine-enkephalin was utilized as the lock mass, a standard practice in mass spectrometry for ensuring mass accuracy throughout the analysis. In the negative ion mode, leucine-enkephalin was set to register a mass-to-charge ratio ([M-H]^−^) of 554.2615. Using a lock mass is essential for maintaining the precision and reliability of the mass spectrometric measurements.

### 2.6 High-performance liquid chromatography analysis

The plant samples of *S. strigillosa, S. baicalensis*, and *S. barbata* were meticulously processed following a method previously described in our earlier study (Shen et al., 2019). Following this established approach, the samples underwent comprehensive analysis per the protocols we have previously detailed (Shen et al., 2019). To ensure robust and reliable data, three biological replicates were conducted, each utilizing different individuals of the same species to account for biological variability.

### 2.7 MIC determination

Following the traditional medicinal parts collected from *Scutellaria* plants, the *S. baicalensis* are categorized into aerial and root parts. For *S. strigillosa* and *S. barbata*, the entire herb is utilized. After drying, the samples are crushed and sieved through a 50-mesh screen. Precisely weigh approximately 5 grams of each powdered sample into a 50 ml volumetric flask, add methanol to the mark, and subject to ultrasonic extraction at room temperature for 1 hour. Filter the solution and return the residue to the volumetric flask. Repeat the extraction and filtration process twice (for three extractions, yielding 150 mL). Combine the extracts and concentrate under reduced pressure. Transfer the concentrated extract to a sample vial using 20% methanol-water solution, and vacuum freeze-dry the extract at -55°C and 0.09 Pa.

*Staphylococcus aureus* and *Pseudomonas aeruginosa* strains were inoculated on LB solid medium and incubated at 37°C for approximately 24 hours for subsequent use. Following the microdilution method recommended by the Clinical and Laboratory Standards Institute (CLSI), the MICs of three species of *Scutellaria* were determined for the experimental strains (*Staphylococcus aureus* and *Pseudomonas aeruginosa*). The bacterial suspension was adjusted to 0.5 McFarland standard using a sterilized CAMHB medium. In a sterile 96-well plate, 194 μL of the bacterial suspension was added to each well, followed by 6 μL of medicinal solutions with 200-0.390625 g/L concentrations. Each drug was tested in triplicate. For the positive control, 100 μL of CAMHB medium was mixed with 100 μL of the bacterial suspension. For the negative control, 194 μL of broth medium was mixed with 6 μL of dimethyl sulfoxide (DMSO). The 96-well plate was placed in a microplate reader to measure the OD600 value and then incubated at 37°C for 20-24 hours. After incubation, the OD600 value was measured again. To eliminate the influence of drug pigments, the inhibition rate was calculated using the formula:


Inhibition rate=[1−(A'−A)/(A'blank−Ablank)]×100%

where A’ and A are the absorbance values of the experimental group after and before 20-24 hours of incubation, respectively; A’blank and Ablank are the absorbance values of the positive control well after and before 20-24 hours of incubation, respectively.

### 2.8 Data analysis

The Progenesis QI 2.3 software (by Waters, Milford, MA, United States) was employed to process centroid MS^E^ raw data from the UPLC-Q-TOF-MS analysis. This comprehensive software suite facilitated various steps in the data processing workflow, including the importation of data, alignment review, setting up the experimental design, selection of peaks, deconvolution, and, ultimately, the identification of metabolites. In addition to the Progenesis QI software, the MetaboAnalyst 6.0 webserver was utilized to conduct multivariate statistical analyses. This analysis was crucial in understanding the complex data set and included partial least squares discriminant analysis (PLS-DA) in the Pareto scaling mode. Furthermore, heatmap analysis was also performed by R 4.3.1. These analytical tools are instrumental in identifying patterns and relationships within the data, providing a comprehensive understanding of the metabolic profiles studied.

## 3 Results

### 3.1 Genomic characteristics of chloroplast of *S. strigillosa*

**Figure 2:**
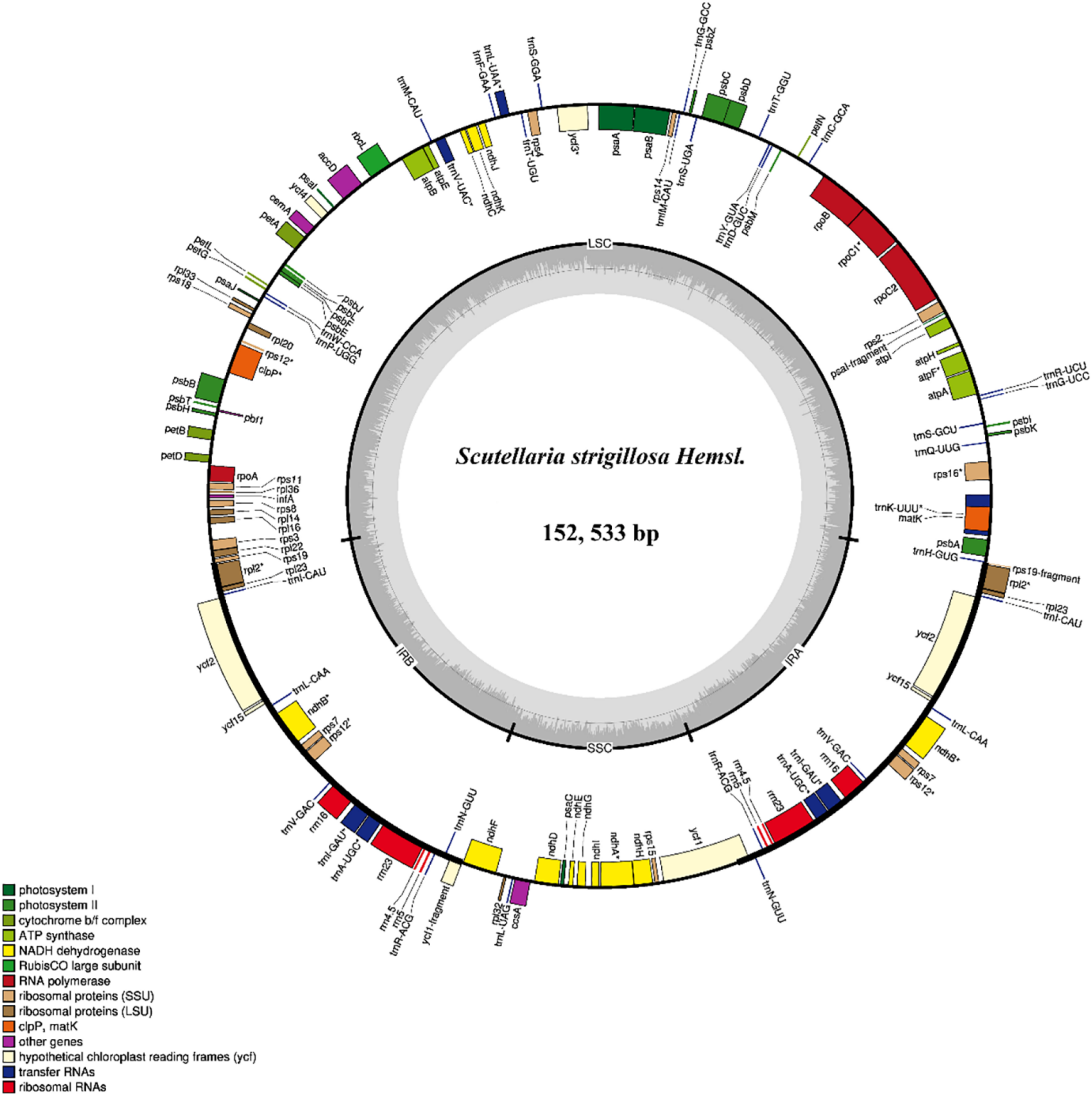
Gene map of the complete chloroplast genomes of *S. strigillosa*. Genes inside the circle are clockwise, while genes outside the circle are counterclockwise. The different colors indicate different functional groups of genes. Dark gray areas inside the circle indicated GC content, while light gray areas indicated AT content of the genome.

Using Illumina HiSeq/MiSeq sequencing platforms, we obtained 3520 M (Illumina raw reads) for *Scutellaria strigillosa* Hemsl. 3508 M reads were finally assembled to generate complete chloroplast genomes. The complete chloroplast genomes of *S. strigillosa*, were determined to be 152,533 bp in total size (**Figure 2**). Its average GC content was 38.3%, similar to other *Scuteelaria* species (Lukas and Novak, 2013; Zhao et al., 2020). The GC content was the highest (43.61%) in the IR regions, the lowest (32.5%) in the SSC region, and 36.34% in the LSC region. The IR region is more conserved than a single copy region in the chloroplast genome of many species, which may be related to the high GC content (Cui et al., 2019; Yan et al., 2019).

In the species of *S. strigillosa*, a comprehensive analysis identified a total of 113 genes within its chloroplast genome. This genetic repertoire included 80 protein-coding genes, 29 tRNA genes, and four rRNA genes. Notably, 18 of these genes were found to be duplicated in the Inverted Repeat (IR) regions of the genome. The duplicated genes comprised seven protein-coding genes (namely *rps12*, *rps7*, *ndhB*, *ycf15*, *ycf2*, *rpl23*, *rpl2*), seven tRNAs (*trnN-GUU, trnR-ACG, trnA-UGC, trnI-GAU, trnV-GAC, trnL-CAA, trnI-CAU*), and four rRNAs (*rrn5*, *rrn 4.5*, *rrn23*, *rrn16*). The distribution of these genes across the chloroplast genomes of *S. strigillosa* was identical. In the Large Single-Copy (LSC) regions, 62 protein-coding genes and 21 tRNA genes were encoded. Meanwhile, the Small Single-Copy (SSC) regions contained 12 protein-coding genes and one tRNA gene.

### 3.2 Interspecific comparison and phylogenetic analysis

The expansion and contraction of IR regions in the chloroplast genomes of *S. strigillosa, S. baicalensis*, *S. barbata* were compared. The complete chloroplast genomes of *S. strigillosa, S. baicalensis*, *S. barbata* were determined to be 151,8817-152,533 bp in total size. The length of *S. strigillosa* was the longest, and *S. baicalensis* was the lowest. The chloroplast genomes all exhibited the typical quadripartite structure with a large single-copy region (LSC) (83,990–84,583 bp), a small single-copy region (SSC) (17,331–17,517 bp), and a pair of inverted repeats (IRs) (25,240–25,248 bp). The expansion and contraction of IR regions in the chloroplast genomes of *Scutellaria* were compared. The results showed that the length of the single-copy region (SC) and the internal size of the genes of *ycf1* that reach the IR region boundary changed, and pseudogenes were produced at the corresponding positions of the IR region. The IRb/LSC junction was located within the rps19 gene in all species. The *rps19* gene had 46 bp projections into the IRa region, resulting in a portion of the *rps19* gene (*rps19* fragment) in the IRb region. The *ycf1* gene straddles the IRa/SSC boundary, but only a tiny fraction of the gene (772 bp-783 bp) is located in the IRb region, and a pseudogene *ycf1* was detected in the IRa region. The length of *trnH* from the IRa/LSC border was 0 bp.

Interspecific comparison of the chloroplast genomes from *S. strigillosa, S. baicalensis*, *S. barbata* was conducted using mVISTA, employing the annotated sequence of *S. baicalensis* as the reference. The mVISTA analysis revealed a high level of sequence conservation across the chloroplast genomes of these *Scutellaria* species, particularly within the protein-coding regions. In contrast, the non-coding regions exhibited greater variability. The variability was notably more pronounced in the LSC and SSC regions than in the IR regions. This pattern of variation is characteristic of chloroplast genomes, where the IR regions tend to be more conserved due to their gene content, which often includes rRNA and tRNA genes, and possibly due to mechanisms that promote sequence correction between the repeats. A nucleotide variability (Pi) analysis further highlighted specific regions with elevated variability. Four regions exhibiting high Pi values (>0.035) were identified as potential hotspots of interspecific variation: *trnK(UUU)*-*rps16*, *trnN*(*GUU*) - *trnR*(*ACG*), *accD*-*psaI, psbE*-*petL*. These regions, which include coding and non-coding DNA, may be particularly interesting for phylogenetic studies or for understanding the evolutionary dynamics within the genus *Scutellaria*. These findings contribute to our understanding of chloroplast genome evolution in *Scutellaria* and may facilitate the development of molecular markers for population genetics, species identification, and phylogenetic studies within this genus.

**Figure 3:**
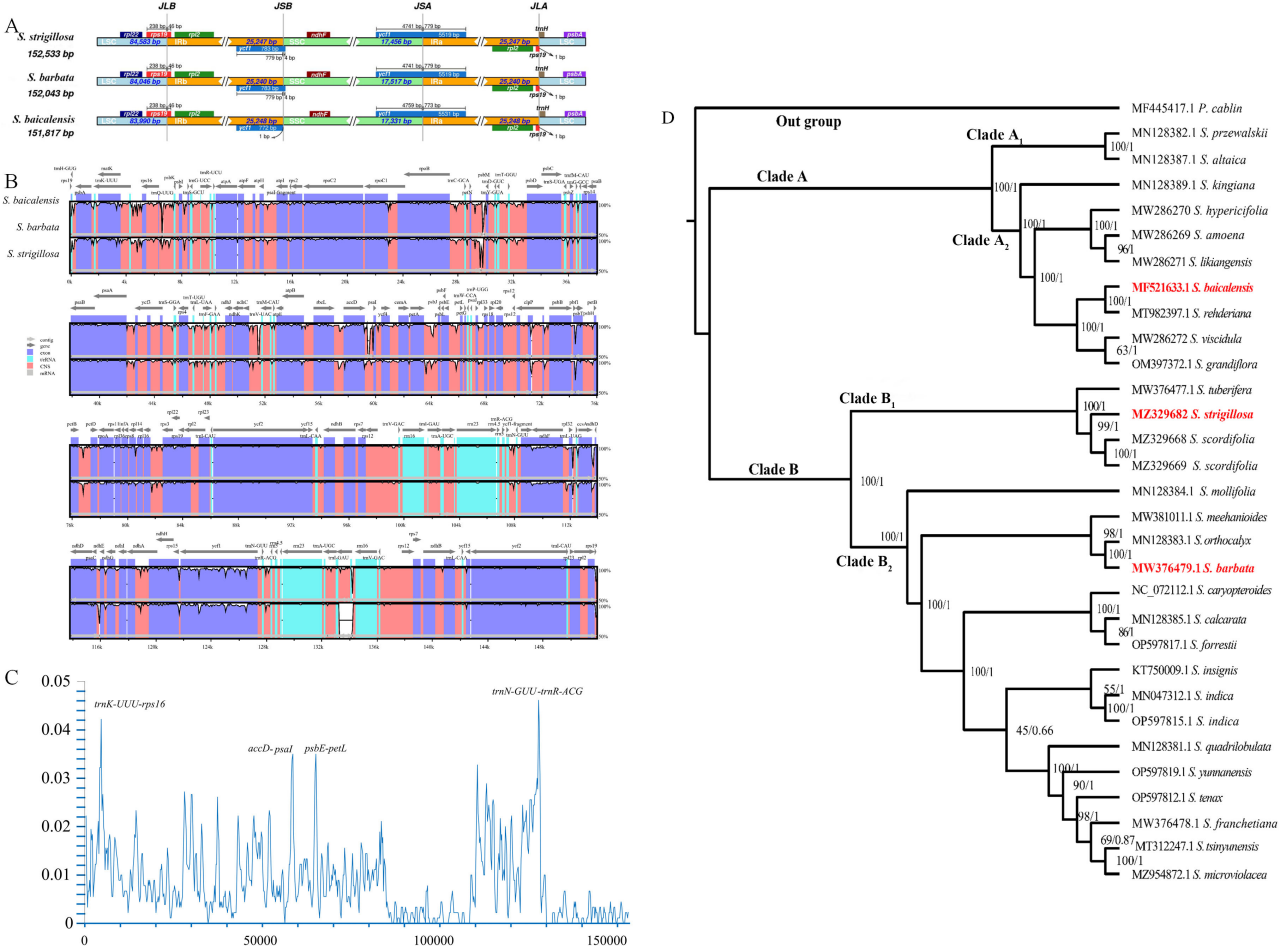
Interspecific comparison and phylogenetic analysis. **(A)** Comparison of LSC, IRb, SSC, and IRa border regions in *S. strigillosa, S. baicalensis*, *S. barbata*. **(B)** Sequence alignment of the whole plastomes of 3 taxa of *Scutellaria* using the Shuffle-LAGAN alignment algorithm in mVISTA, with *Scutellaria baicalensis* as the reference. **(C)** Sliding window analysis of the chloroplast genomes of *S. strigillosa, S. baicalensis*, and *S. barbata*. Window length: 600 bp; step size: 200 bp. X-axis: position of the midpoint of a window. Y-axis: nucleotide. **(D)** Phylogenetic relationships of the 28 species by ML and BI analyses (Pogostemon cablin as an outgroup) based on alignments of complete chloroplast genomes. The numbers above nodes are supported values with ML bootstrap values on the left and Bayesian posterior probability (PP) values on the right.

In this study, based on complete chloroplast genome sequences, 28 species from *Scutellaria* were used to construct an ML tree and BI trees (**Figure 3**). All species of *Scutellaria* were classified into two subclades (ML/BS 100, BI/PP 1), namely Subclade A (100, 1) and Subclade B (100, 1). Subclade A (100, 1) comprised ten species and was further divided into Subclade A1 and Subclade A_2_, both supported with 100%. Subclade A_1_ contains two species, *S. altaica* Ledeb. ex Sweet and *S. przewalskii* Juz. Subclade A_2_ consists of six species of *S. kingiana* Prain, S. *hypericifolia* H. Lév.*, S. amoena* C. H. Wright*, S. likiangensis* Diels*, S. baicalensis, S. rehderiana* Diels*, S. viscidula* Bge., and *S. grandiflora* Sims. In the maximum likelihood tree constructed based on the chloroplast genome, *S. kingian*a showed strong support (100, 1) for clustering with these six species, indicating a close affinity. Subclade B (100, 1) consists of nineteen species, including Subclade B_1_ and Subclade B_2_, both support with 100% support. Subclade B_1_ comprised *S. tuberifera* C. Y. Wu & C. Chen, *S. strigillosa*, and *S. scordifolia*. Notably, in the flora of China, *S. tuberifera* belongs to Subgen. Scutellariopsis. The species of *S. strigillosa* and *S. scordifolia* belong to Ser. Scordifoliae, Sect. Galericularia, Subg. *Scutellaria*. However, in the phylogenetic tree based on the chloroplast genome, they exhibited distant relationships with Sect. Galericularia, with them being closely related to *S. tuberifera.* Subclade B_2_ comprises sixteen other species, and the results are similar to the previous report (Shen et al., 2022). In addition, as shown in the phylogenetic tree, compared with *S. baicalensis*, *S. strigillosa* was more closely related to *S. barbata*.

### 3.3 Specialized metabolites in the *Scutellaria* species

The UPLC-Q-TOF-MS analysis of the whole plant of *S. strigillosa, S. baicalensis*, and *S. barbata* was conducted in negative ion mode, utilizing electrospray ionization (ESI^−^). A Principal Component Analysis (PCA) was initially undertaken to explore the chemical diversity among the tested *Scutellaria* species, as illustrated in **Figure 3**. This analysis revealed distinct variations in the chemical compositions of the three *Scutellaria* species. Notably, the chemical profiles of *S. strigillosa* showed a closer clustering to *S. barbata* than *S. baicalensis*. This finding aligns with the results from the phylogenetic tree analysis, which indicates that *S. strigillosa* and *S. barbata* are closely related and share similar chemical compositions. Furthermore, a marked difference was observed in the chemical composition of the root of *S. baicalensis*, which significantly diverged from its aerial parts and the other studied species.

Progenesis QI software is widely recognized for its reliability and frequent use for metabolomic data online identification, as cited in sources (Gu et al., 2019; Wang et al., 2019). In our research, the tentative identification of these three *Scutellaria* species chemical constituents was achieved through an in-house *Scutellaria* genus library search and Progenesis MetaScope’s internal database *via* Progenesis QI, supplemented by literature and standard comparisons (refer to **Table 1**). This process involved matching m/z values and characteristic fragment ions against potential metabolites in various databases. For instance, the online identification *via* Progenesis QI pinpointed baicalin using its molecular weight and adduct ion (m/z 467.0583, M+Na-2H), along with a typical fragment ion (m/z 269.0431, C_15_H_9_O_5_^-^). Isocarthamidin-7-*O*-*D*-glucuronide was recognized by two adduct ions, M-H^-^ (C_21_H_19_O_12_^-^, m/z 463.0865) with a fragment ion identified as C_15_H_11_O_6_^-^ (m/z 287.0555). Metabolite 5,7,4’-trihydroxyflavanone (naringenin, m/z 287.054) with a serial fragment ion identified as C_14_H_12_O_5_-H (m/z 259.0612), C_8_H_6_O_5_-H (m/z 181.0135), C_8_H_6_O- (m/z 119.0494). Metabolite 6’-O-E-p-coumaroylgardoside (m/z 487.1232) along with the typical fragment ions C_12_H_12_O_6_-H_2_O-H (m/z 233.0468), C_9_H_8_O_2_- (m/z 149.0574), C_7_H_6_O_2_- (m/z 149.0574). Reference substances further supported the reliability of Progenesis QI identifications. In negative ion mode, most metabolites exhibited deprotonation, commonly resulting in -H_2_O and -CO_2_ losses. Carboxylic acid-containing metabolites typically lose CO_2_. Flavonoid glycosides were predominantly identified through specific molecular losses, such as CO, and cleavages *via* the RDA reaction. Sugar losses, notably arabinose, and glucuronide, were mainly observed in flavonoid glycosides. Similar to other *Scutellaria* species, *S. strigillosa* contains unique metabolites, including numerous isomers with similar molecular weights, mass spectrometry fragmentation patterns, and subtle retention time differences, posing challenges in distinguishing structural isomers using solely mass spectrometry-based metabolomics. Consequently, metabolite identification with Progenesis QI involved assessing polarity, ion fragmentation patterns, and UV absorption wavelengths, with literature references for further verification. A total of 112 metabolites were tentatively identified in negative ion mode using this methodology. Of these, 16 metabolites corresponded with the standard comparison results identified by Progenesis QI (**Table 1**). These included 3 alkaloids,1 *α-*pyrone glycoside, 30 diterpenoids, 56 flavonoids, 15 iridoid glycoside,2 phenolics, 4 phenylethanoid glycosides, and 1 other metabolite.

**Table 1:** Metabolites putatively identified from the *S. strigillosa,S. baicalensis* and *S. barbata*.

No.	*tR* (Min)-m/z(n)	Adducts	Molecular Formula	Mass Error (ppm)	Metabolite type	Key MS*^E^* fragment ions (Da)	Identification
1	2.16_300.1200n	M+Cl, M+FA-H	C_14_H_20_O_7_	-3.0200	Iridoid glycoside	335.0864, 315.1055	4-Hydroxy-*β*-Phenylethyl-*β-D-*Glucopyranoside
2	2.23_383.0962m/z	M+Na-2H	C_15_H_22_O_10_	0.7285	Iridoid Glycoside	315.1015, 291.1058	Catalpol
3	2.66_465.1018m/z	M-H	C_21_H_22_O_12_	-4.3544	Alpha-pyrone glycoside	465.1032, 437.1078, 339.0698, 125.0235	Scusalvioside B
4	2.89_375.1278m/z	M-H	C_16_H_24_O_10_	-4.9113	Iridoid glycoside	375.1298, 213.0758, 151.0754	8-*Epi*-Loganic Acid
5	3.15_553.1559m/z	M+FA-H	C_24_H_28_O_12_	-0.8223	Iridoid glycoside	433.1155, 325.0910135.0441	6’-*O*-*p*-E-Coumaroyl-8-*Epi*-Loganic Acid
6	3.42_385.1116m/z	M+Na-2H	C_15_H_24_O_10_	-0.1499	Iridoid glycoside	325.0310, 151.0390, 125.0237	Dihydrocatalpol
7	3.42_415.0639m/z	M+K-2H	C_15_H_22_O_11_	-2.4359	Iridoid glycoside	325.0310, 151.0390, 125.0237	Macfadienoside
8	3.61_609.2006m/z	M+Cl	C_32_H_34_N_2_O_8_	-0.5133	Alkaloids	475.1809, 429.1383	7*-O-*Nicotinoylscutebarbatine H
9	3.65_476.1880n	M-H, M+Cl, M+K-2H	C_21_H_32_O_12_	-2.9521	Phenylethanoid glycoside	475.1315, 385.1119, 329.1221, 323.1322, 209.0819	Darendoside B
10	3.75_595.2550m/z	M+FA-H	C_32_H_38_O_8_	0.2834	Diterpene	547.2320, 193.0489	Barbatin F
11	3.92_593.1506m/z	M+FA-H	C_26_H_28_O_13_	-1.0763	Flavonoid	593.1505, 473.1081, 411.1034, 195.0749	Chrysin-6*-C-L-*Ara-8*-C-D-*Glucopyranside
12	4.04_301.0340m/z	M-H	C_15_H_10_O_7_	-4.6367	Flavonoid	175.0026, 119.0124	3,5,7,2’6’pentahydroxyflavone
13	4.11_515.2304m/z	M+FA-H	C_27_H_34_O_7_	3.6460	Diterpene	469.2259, 411.1384, 381.1783	Scutellone D
14	4.53_461.0715m/z	M-H	C_21_H_18_O_12_	-2.3751	Flavonoid	285.04	Kaempferol 3-Glucuronide
15	4.53_464.0958n	M-H	C_21_H_20_O_12_	0.6442	Flavonoid	435.0933, 287.0559, 285.0401, 269.0461, 175.0428	Isocarthamidin-7*-O-D-*Glucuronide*
16	4.57_547.1459m/z	M-H_2_O-H	C_26_H_30_O_14_	0.2416	Flavonoid	529.1331, 427.1028, 397.0923, 379.0324, 347.0705, 337.0717	Naringenin 7*-O-*(2-*β-D-*Apiofuranosyl)- *β-D-*Glucopyranos
17	4.70_567.1970m/z	M+K-2H	C_29_H_38_O_9_	-6.0388	Diterpene	419.1167, 355.0790, 197.0480	Scutellone C
18	4.87_287.0548m/z	M-H	C_15_H_12_O_6_	-4.4571	Flavonoid	259.0602, 181.0135, 161.0241, 119.0494	5,7,4’trihydroxyflavanone (Naringenin)
19	4.87_464.0953n	M-H	C_21_H_20_O_12_	-0.2977	Flavonoid	435.0933, 287.0559, 285.0401, 269.0461, 175.0428	Carthamidin-7*-O-D-*Glucuronide*
20	4.87_623.1971m/z	M-H	C_29_H_36_O_15_	-1.6067	Phenylethanoid glycoside	623.1981, 547.1461, 487.1236, 457.1131, 161.0242, 135.0442	Acetoside
21	4.90_529.1325m/z	M+Na-2H	C_24_H_28_O_12_	-0.4489	Iridoid glycoside	473.1079, 297.0759	Scutellarioside II
22	5.13_462.0790n	M-H, 2M-H	C_21_H_18_O_12_	-1.7946	Flavonoid	285.0401, 283.0245, 175.0268	Scutellarin*
23	5.13_537.1593m/z	M+FA-H	C_24_H_28_O_11_	-4.1901	Iridoid glycoside	493.1409, 417.1046, 113.0287	Scutellarioside I
24	5.16_286.0468n	M-H, 2M-H	C_15_H_10_O_6_	-3.2735	Flavonoid	271.0248, 242.0290, 135.0074,	5,7,2’5’tetrahydroxyflavone
25	5.33_448.1006n	M-H, M+Na-2H	C_21_H_20_O_11_	0.1895	Flavonoid	433.1134, 309.0370, 151.0034	Luteolin-7*-O-D-*Glucopyranside*
26	5.36_547.1453m/z	M-H	C_26_H_28_O_13_	-0.8162	Flavonoid	429.1132, 547.1137, 527.1030, 367.0820	Chrysin 6*-C-α-L-*Arabinopyranoside-8*-C-*Glucoside
27	5.43_589.2419m/z	M+K-2H	C_29_H_44_O_10_	-0.3241	Diterpene	495.2980, 251.1269	Scutelaterin C
28	5.52_497.2198m/z	M+FA-H	C_27_H_32_O_6_	3.8439	Diterpene	425.1993, 387.1426	Scutellone E
29	5.59_582.1940n	M-H, 2M-H	C_27_H_34_O_14_	-1.4755	Flavonoid	581.1871, 461.1481, 299.0917	Amoenin A
30	5.62_483.0532m/z	M+Na-2H	C_21_H_18_O_12_	-2.7609	Flavonoid	285.0396, 151.0388	Luteolin-7*-O-D-*Glucuronopyranoside
31	5.65_431.0979m/z	M-H_2_O-H	C_21_H_22_O_11_	-0.9867	Flavonoid	301.0708, 141.0188, 111.0178	(*cis*)-5,7,2’Trihydroxyflavanonol-3*-O-β-D-*Glucopyranoside
32	5.65_499.0847m/z	M+Na-2H	C_22_H_22_O_12_	-2.2508	Flavonoid	477.1034, 301.0708, 111.0078,	5,7,2’Trihydroxy-6-Methoxyflavanone-7*-O-β-D-*Glucuronopyranoside
33	5.72_547.1445m/z	M-H	C_26_H_28_O_13_	-2.1354	Flavonoid	457.1138, 439.0932, 379.0824, 145.0295	Chrysin-6*-C-D-*Glu-8*-C-L-*Arabinopyranoside
34	5.76_591.1692m/z	M+FA-H	C_27_H_30_O_12_	-5.0318	Flavonoid	455.0968, 205.0492	Scuregelioside B
35	5.79_551.1753m/z	M+FA-H	C_25_H_30_O_11_	-3.3220	Phenylethanoid glycoside	505.1752, 297.0952, 235.0605, 191.0680,151.0754	2-(3’Hydroxy-4’methoxyphenyl)-Ethyl-1*-O-β-D-*(4*-D-*Feruolyl)-Glucoside
36	5.89_409.0926m/z	M+Cl	C_16_H_22_O_10_	4.9955	Iridoid glycoside	373.1153, 319.0599, 291.0295, 239.0345, 199.0386, 163.0400.	Gardoside
37	6.01_487.1232m/z	M-H_2_O-H	C_24_H_26_O_12_	-2.8023	Iridoid glycoside	233.0468, 149.0574, 107.0493,	6’-O-*E*-*p*-Coumaroylgardoside
38	6.25_301.0705m/z	M-H	C_16_H_14_O_6_	-4.0548	Flavonoid	301.0692, 195.0323, 119.0490	5,7,4’Trihydroxy-6-Methoxyflavanone
39	6.28_559.1406m/z	M+Na-2H	C_25_H_30_O_13_	-4.9781	Iridoid Glycoside	505.1718, 417.1276, 209.0706	Picroside III
40	6.38_652.2351n	M-H, M+Cl	C_31_H_40_O_15_	-2.4468	Iridoid Glycoside	651.2286, 497.1588, 475.1793, 175.0394	Isomartynoside
41	6.38_673.2141m/z	M+Na-2H	C_31_H_40_O_15_	4.1969	Iridoid Glycoside	271.0604, 243.0654, 173.0599	Martynoside
42	6.47_448.0993n	M-H	C_21_H_20_O_11_	-2.7744	Flavonoid	447.0929, 271.0604, 193.0403, 175.0247	5,7,2’-Trihydroxyflavone 2’*-O-β-D-*Glucuronopyranoside
43	6.54_446.0841n	M-H, M+Cl	C_21_H_18_O_11_	-1.8896	Flavonoid	269.0453	Baicalin*
44	6.54_417.0833m/z	M+K-2H	C_15_H_24_O_11_	7.3307	Iridoid Glycoside	267.0295, 239.0347, 71.0131	10-Descinnamoylglobularinin
45	6.61_673.2837m/z	M+FA-H	C_34_H_44_O_11_	-4.5657	Diterpene	583.2568, 521.1782, 377.1578, 165.0181	Scutebarbolide E
46	6.64_286.0469n	M-H, M+K-2H	C_15_H_10_O_6_	-3.0714	Flavonoid	257.0492, 137.0204, 117.0335	Scutellarein*
47	6.64_333.0601m/z	M+FA-H	C_15_H_12_O_6_	-5.1318	Flavonoid	261.0724, 241.0493, 147.0078	Dihydroscutellarein
48	6.68_485.1048m/z	M+Na-2H	C_22_H_24_O_11_	-3.8257	Flavonoid	373.0924, 113.0238, 95.0131	Scuteamoenoside
49	6.71_314.0787n	M-H_2_O-H, M+Na-2H	C_17_H_14_O_6_	-0.9557	Flavonoid	295.0614, 279.0647, 241.0493, 145.0287	5,7,2’5’Tetrahydroxy-8,6’dimethoxyflavone
50	6.71_415.1023m/z	M-H	C_21_H_20_O_9_	-2.8045	Flavonoid	295.0611, 267.0657	Chrysin-8*-C-D-*Glucopyranside
51	6.71_555.2058m/z	M+Na-2H	C_30_H_34_N_2_O_7_	-10.2502	Diterpene	467.1885, 377.1578, 359.1477, 145.0287	Scutebarbatine N
52	6.71_667.2310m/z	M+Cl	C_36_H_40_O_10_	-0.7892	Diterpene	595.1678, 563.2046, 373.1254	Scutebata A
53	6.78_462.0795n	M-H, 2M-H	C_21_H_18_O_12_	-0.7808	Flavonoid	461.0727, 399.0773, 299.0553, 285.0397, 119.0487	Isoscutellarein 8-glucuronide*
54	6.81_476.0956n	M-H, 2M-H, M-H2O-H	C_22_H_20_O_12_	0.3197	Flavonoid	475.0897, 459.0935, 299.0548, 133.0284	Hispidulin-7*-O-D-*Glucuronide
55	7.00_427.0650m/z	M-H_2_O-H	C_21_H_18_O_11_	-4.6868	Flavonoid	351.0426, 336.0251, 96.9925	Norwogonin-7*-O-D-*Glucuronopyranoside
56	7.03_507.2217m/z	M+FA-H	C_25_H_34_O_8_	-4.0227	Diterpene	507.2215, 453.1719,	Scutefolide D
57	7.07_443.0607m/z	M-H_2_O-H	C_21_H_18_O_12_	-2.7544	Flavonoid	291.0299, 269.0449, 161.0245, 159.0294, 145.0287	Scutellarein-7*-O-D-*Glucopyranside
58	7.07_446.0838n	M-H, M+Na-2H, 2M-H	C_21_H_18_O_11_	-2.5635	Flavonoid	269.0451	Apigenin-7*-O-*Glucuronide
59	7.10_659.1966m/z	M+Na-2H	C_30_H_38_O_15_	1.3382	Phenylethanoid glycoside	573.1613, 269.0449, 199.0390, 171.0437	Leucosceptoside A
60	7.14_301.0704m/z	M-H	C_16_H_14_O_6_	-4.6149	Flavonoid	301.0717, 255.0639, 193.0479	Scuteamoenin
61	7.24_476.0942n	M-H, M-H_2_O-H	C_22_H_20_O_12_	-2.6573	Flavonoid	429.0827, 339.0358, 299.0551,	5,7,2’Trihydroxy-6-Methoxyflavone-7*-O-D-*Glucuronopyranoside
62	7.24_859.1716m/z	2M-H	C_21_H_18_O_10_	-1.3168	Flavonoid	253.0506, 175.0245, 113.0239	Chrysin-7*-O-D-*Glucuronopyranoside*
63	7.27_517.1710m/z	M-H_2_O-H	C_26_H_32_O_12_	-0.9129	Iridoid glycoside	291.1192, 229.0492, 275.0753, 113.0237	6’-*O*-*E*-Caffeoyl- 8 -*Epi*-Loganic Acid
64	7.30_445.1128m/z	M-H	C_22_H_22_O_10_	-2.7831	Flavonoid	445.1127, 373.0924, 113.0237	Acacetin-7*-O-D-*Glucoside (Tilianin)
65	7.30_513.0991m/z	M+Na-2H	C_23_H_24_O_12_	-4.7107	Flavonoid	449.1067, 425.0872, 261.0408, 113.0237	5,2’6’Trihydroxy-6,7-Dimethoxyflavone-2’O*-D-*Glucoside
66	7.46_467.2126m/z	M-H	C_27_H_32_O_7_	10.8580	Alkaloids	421.2073, 97.0287	Scutebarbatine H
67	7.53_446.0838n	M-H, M+Na-2H, 2M-H	C_21_H_18_O_11_	-2.3842	Flavonoid	445.0775, 283.0609, 268.0373, 175.0245, 113.0238	5,7,2’Trihydroxyflavone-7*-O-D-*Glucuronopyranoside
68	7.56_283.0600m/z	M-H	C_16_H_12_O_5_	-4.1580	Flavonoid	268.0370, 163.0023, 119.0126	Acacetin
69	7.56_459.0930m/z	M-H	C_22_H_20_O_11_	-0.5187	Flavonoid	445.0775, 283.0609, 175.0245, 113.0238	Wogonoside*
70	7.60_527.0796m/z	M+Na-2H	C_23_H_22_O_13_	-2.2434	Flavonoid	466.0509, 358.0691, 268.0370, 175.0248,113.0244	Visciduli II-2’O*-D-*Glucuronide
71	7.63_329.0658m/z	M-H	C_17_H_14_O_7_	-2.7746	Flavonoid	314.043	4’,5,7-Trihydroxy-3,6-Dimethoxyflavone
72	7.70_501.0995m/z	M-H2O-H	C_24_H_24_O_13_	-8.3014	Flavonoid	489.1034, 443.0983	5,2’Dihydroxy-7,8,6’trimethoxyflavone-2’-O*-D-*Glucuronopyranoside
73	7.73_489.1030m/z	M-H	C_23_H_22_O_12_	-1.8161	Flavonoid	443.0983, 313.0711	5,7-Dihydroxy-8,2’dimethoxyflavone-7*-O-D-*Glucuronide
74	7.80_555.2353m/z	M+Cl	C_28_H_40_O_9_	-2.5416	Diterpene	453.1372, 291.1200, 249.0519	Scutebarbolide J
75	7.83_446.0834n	M-H, 2M+Hac-H	C_21_H_18_O_11_	-3.3017	Flavonoid	323.0549, 291.0263, 141.0102	Norwogonin-8*-O-D-*Glucuronopyranoside
76	7.90_333.2047m/z	M-H	C_20_H_30_O_4_	-7.1917	Diterpene	303.1938, 287.1997	Scutebarbolide G
77	7.99_225.0557m/z	M+Na-2H	C_14_H_14_O_44_	11.8945	Phenolic	183.0437	Piceatannol
78	8.02_539.2396m/z	M+Cl	C_28_H_40_O_8_	-4.1263	Diterpene	495.2493	Scutefolide C
79	8.12_270.0519n	2M-H, 3M-H	C_15_H_10_O_5_	-3.3025	Flavonoid	225.0558, 211.0394, 163.0392	Apigenin*
80	8.22_203.0704m/z	M-H	C_12_H_12_O_3_	-4.7533	Phenolic	147.0441, 103.0544, 97.0286	(*S*)-2-(4-Hydroxyphenyl)-6-Methyl-2,3-Dihydro-4H-Pyran-4-One
81	8.22_297.0743m/z	M-H	C_17_H_14_O_5_	-8.4356	Flavonoid	147.0441, 103.0545	5-Hydroxy-7,8-Dimethoxyflavone
82	8.33_389.0871m/z	M+FA-H	C_18_H_16_O_7_	-1.9081	Flavonoid	295.0613, 279.0642, 277.0498	5,7-Dihydroxy-8,2’6’trimethoxyflavone
83	8.55_270.0530n	M+FA-H, 2M-H	C_15_H_10_O_5_	0.5370	Flavonoid	269.0453, 251.0343, 223.0400, 123.0087, 101.0384	Baicalein*
84	8.55_288.0635n	M-H_2_O-H	C_15_H_12_O_6_	0.5035	Flavonoid	269.0450, 251.0350, 239.0349, 223.0401	(2r,3r)3,5,7,2’tetrahydroxyflavanone
85	8.65_491.2055m/z	M+Na-2H	C_27_H_34_O_7_	0.8360	Diterpene	469.2232, 153.0549	Scutebarbolide K
86	9.04_411.1801m/z	M+Na-2H	C_22_H_30_O_6_	3.1103	Diterpene	369.1320, 313.1309, 231.1003, 211.1330	6-Acetoxybarbatin C
87	9.38_283.0606m/z	M-H	C_16_H_12_O_5_	-1.9451	Flavonoid	239.0343, 211.0393, 163.0029	Wogonin*
88	9.44_360.0834n	M-H, M+Na-2H	C_18_H_16_O_8_	-2.9944	Flavonoid	313.0719	6,2’-Dihydroxy-5,7,8,6’tetramethoxyflavone
89	9.54_493.2424m/z	M-H	C_26_H_38_O_9_	-3.9411	Diterpene	465.2130, 419.2118, 397.2181, 251.1252	Scutorientalin C
90	9.57_621.2468m/z	M+FA-H	C_32_H_36_N_2_O_8_	2.4915	Alkaloid Glycoside	413.1398	6*-O-*Nicotinoylscutebarbatine G
91	9.71_254.0570n	M-H	C_15_H_10_O_4_	-3.4652	Flavonoid	253.0504, 209.0606, 143.0495	Chrysin*
92	9.71_365.0633m/z	M+Na-2H	C_18_H_16_O_7_	-2.7183	Flavonoid	351.0472, 145.0287, 101.0387	Tenaxin-I
93	9.77_284.0676n	M-H, 2M-H	C_16_H_12_O_5_	-3.1051	Flavonoid	223.0307, 211.0392, 195.0446, 184.0519, 163.0024, 139.0545, 110.0002	Oroxylin A*
94	9.88_313.0706m/z	M-H_2_O-H	C_17_H_16_O_7_	-3.4052	Flavonoid	298.0475, 185.0235	Dihydrorehderianin I
95	9.88_381.0577m/z	M+Na-2H	C_18_H_16_O_8_	-3.9979	Flavonoid	313.0717, 213.0212, 185.0235	5,6,2’-Trihydroxy-7,8,6’trimethoxyflavone
96	10.13_435.2024m/z	M+FA-H	C_22_H_30_O_6_	-0.1494	Diterpene	313.1769, 299.1637	Scutebata I
97	10.23_343.0815m/z	M-H	C_18_H_16_O_7_	-2.5417	Flavonoid	328.0578, 313.0350, 270.0182	Eupatilin
98	10.42_558.2371n	M-H, M+FA-H	C_32_H_34_N_2_O_7_	0.9646	Diterpene	557.2291, 289.1775	Scutebata U
99	10.42_565.2653m/z	M+FA-H	C_28_H_40_O_9_	-0.2955	Diterpene	407.2031, 265.1420, 157.0490, 113.0603	Scutebata R
100	10.46_598.2655m/z	M+FA-H	C_31_H_39_NO_8_	-0.4178	Diterpene	475.2352, 289.1775	Scutelinquanines C
101	10.49_491.2292m/z	M+FA-H	C_25_H_34_O_7_	1.1889	Diterpene	475.2352, 331.1894, 289.1775	Scutebarbolide I
102	10.59_533.2370m/z	M+Cl	C_29_H_38_O_7_	11.6979	Diterpene	553.2260, 493.2445	Scutellone H
103	10.73_459.2007m/z	M+Na-2H	C_23_H_34_O_8_	1.4516	Diterpene	416.1478, 345.1694, 199.1316	Scutaltisin E
104	10.95_387.1796m/z	M-H2O-H	C_22_H_30_O_7_	-4.2135	Diterpene	361.1649	Scutalbin A
105	10.95_520.2675n	M-H	C_28_H_40_O_9_	0.5273	Diterpene	519.2960, 395.1750, 361.1649, 333.2031, 223.0942,	Scutolide G
106	11.12_317.2107m/z	M-H	C_20_H_30_O_3_	-4.8225	Diterpene	291.1951, 273.1381, 257.1484, 181.1124	Scubatine B
107	11.19_329.1744m/z	M-H_2_O-H	C_20_H_28_O_5_	-4.2468	Diterpene	329.1759, 285.1497, 139.1119	Barbatin C
108	11.19_461.2172m/z	M-H	C_25_H_34_O_8_	-1.9605	Diterpene	329.1736, 197.1173	Scutefolide A
109	11.29_506.2510n	M+FA-H, 2M-H	C_27_H_38_O_9_	-1.1958	Diterpene	445.2231, 277.1445, 115.0390	Scutebarbolide B
110	11.29_546.2814n	M+FA-H, 2M+FA-H	C_30_H_42_O_9_	-2.7618	Diterpene	445.2188, 223.0941, 115.0390	Scutefolide K
111	11.68_519.2162m/z	M+Cl	C_28_H_36_O_7_	1.5019	Diterpene	399.1942, 245.1710, 121.0288	Scutellone I
112	11.82_472.3536n	M-H	C_30_H_48_O_4_	-3.4151	other	471.3473, 539.3345	Pygenic Acid a

*Identifications were confirmed by comparing t_R_ and MS spectra to standard metabolites.

**Figure 4:**
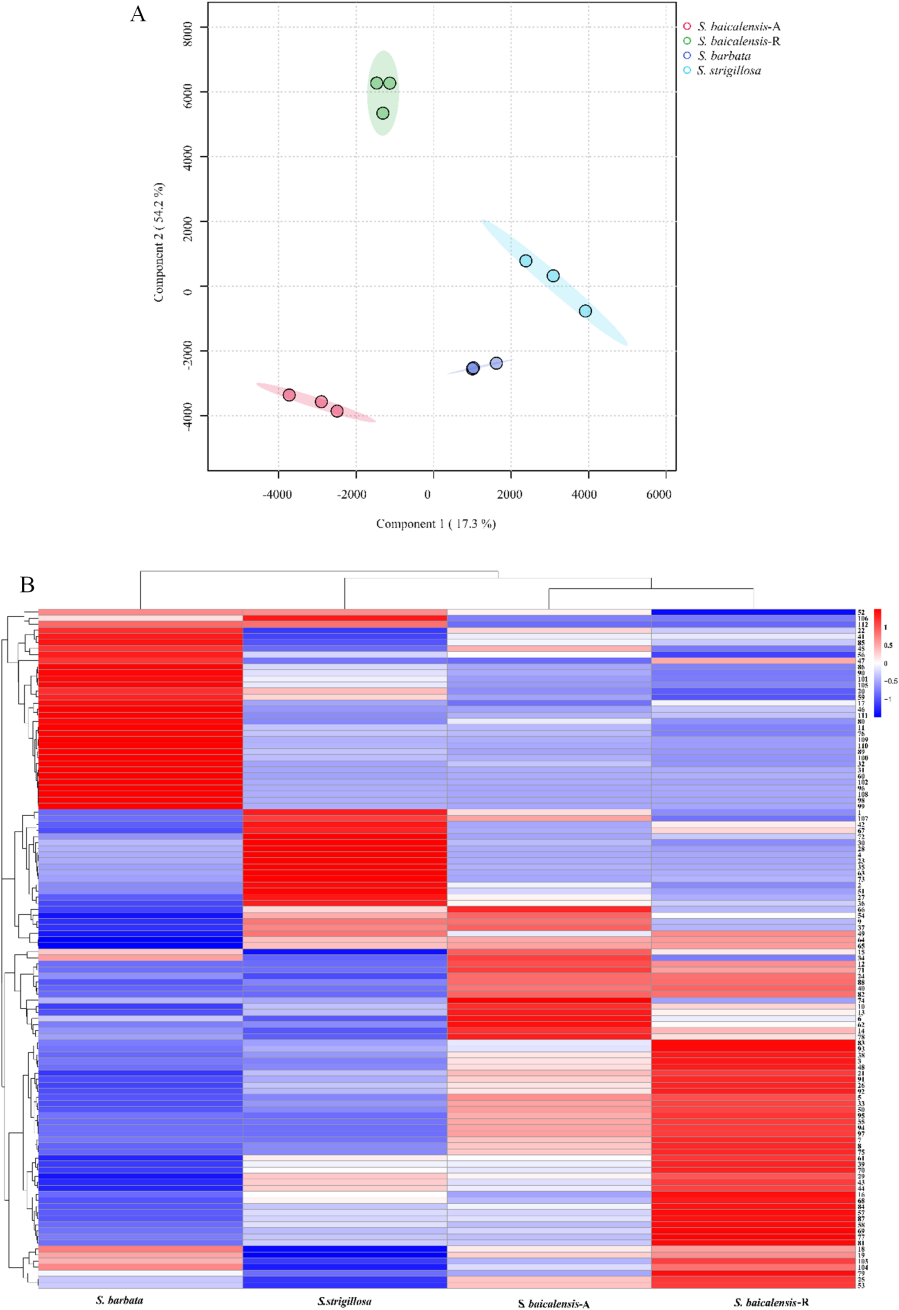
The multivariate analysis of the metabolite data was derived from *S. strigillosa*, *S. baicalensis*, and *S. barbata*. **(A)** PCA score map **(B)** Clustering heatmap of 112 metabolites in different species (the number of analytes corresponds to **Table 1**).

A comprehensive heatmap analysis assessed the relative abundances of 112 distinct metabolites identified in these three *Scutellaria* species (**Figure 4**). This analysis revealed a fascinating profile of phytochemicals within the plant. Notably, certain metabolites demonstrated particularly high concentrations in different species. The heatmap analysis highlighted the distinct metabolite profiles among the *Scutellaria* species and revealed intricate patterns of phytochemical variation within each species. For instance, *S. strigillosa, S. baicalensis* and *S. barbata* displayed a rich diversity in their metabolite profiles, and certain metabolites were exclusively present or significantly enriched in one part of the plant compared to the other. In *S. strigillosa*, compounds 1, 2, 4, 27, 28, 30, 35, 36, 42, 49, 51, 63, 67, 72, 73, 106, and 107 were highly abundant. In contrast, the aerial part of *S. baicalensis* predominantly contains compounds 6, 9, 10, 14, 15, 18, 19, 22, 25, 34, 45, 54, 62, 66, 74, 78, and 85, all exhibiting relatively higher concentrations. *S. barbata* is characterized by a high relative abundance of compounds 11, 17, 20, 22, 31, 32, 41, 45-47, 56, 59, 60, 76, 80, 85, 86, 89, 90, 96, 98-102, 105, 108-112. Lastly, the root of *S. baicalensis* shows a significant presence of compounds 3, 5, 7, 8, 16, 18, 19, 21, 25, 26, 29, 33, 38, 39, 43, 44, 48, 50, 53, 55, 57, 58, 61, 68-70, 75, 77, 79, 81, 83, 84, 87, 91-95, 97, 103, and 104. This suggests a complex biosynthetic pathway that might be influenced by different genetic predispositions. Furthermore, intriguing clusters of shared metabolites across the species were identified upon closer inspection of the heatmap. Notably, metabolites such as 29 (amoenin A), 43 (baicalin), 44 (10-descinnamoylglobularinin), 64 (acacetin-7-*O*-*D*-glucoside), and 65 (5,2’,6’Trihydroxy-6,7-dimethoxyflavone-2’ *O*-*D*-glucoside) were found to have a relatively high concentration in both *S. strigillosa* and the root of *S. baicalensis*. Metabolites 52, 102, 105, 106, and 112 also exhibited a high presence in both *S. strigillosa* and *S. barbata*. These observations suggest potential evolutionary or functional similarities among these species. Several compounds, such as isocarthamidin-7-*O*-*D*-Glucuronide (15), carthamidin-7-*O*-*D*-Glucuronide (19), scutellarin (22), baicalin (43), scutellarein (46), isoscutellarein 8-glucuronide (53), chrysin-7-*O*-*D*-glucuronopyranoside (62), wogonoside (69), baicalein (83), wogonin (87), seemed to be ubiquitous across the genus. This prevalence underscores their essential role in the biology of these plants. The comprehensive heatmap analysis provided a deeper understanding of the phytochemical diversity within the Scutellaria genus and laid a foundation for further studies on these compounds’ ecological and pharmacological significance. The insights gained from this study could pave the way for developing new drugs and therapies, leveraging the unique chemical compositions of these medicinal plants.

### 3.4 Quantitative determination of the 12 assessed chemical components

The HPLC analytical method described in our previous reports (Shen et al., 2019) was used to quantify 12 compounds in *S. strigillosa*, *S. baicalensis*, and *S. barbata*. These compounds were selected for quantification due to their significant contributions to the medicinal properties of *Scutellaria* species. Compounds such as isocarthamidin-7-*O*-*D*-glucuronide (15), carthamidin-7-*O*-*D*-glucuronide (19), scutellarin (22), isoscutellarein 8-glucuronide (53), chrysin-7-*O*-*D*-glucuronopyranoside (62), and apigenin (79) are abundant in the aerial parts of *Scutellaria* species and are known for their anti-inflammatory, antioxidant, neuroprotective, and antibacterial activities (Quan et al., 2023; Shen et al., 2021, 2022). Flavones lacking a 4′-OH group on the B-rings, such as baicalin (43), wogonoside (69), baicalein (83), wogonin (87), chrysin (91), and oroxylin A (93) are enrichened in the root of *Scutellaria* species (Song et al., 2020; Sun et al., 2020; Zhao et al., 2018) and well-documented for their antibacterial activities, antitumor, anti-inflammatory, antioxidant, hepatoprotective and neuroprotective effects (Shen et al., 2021, 2022).

**Figure 5:**
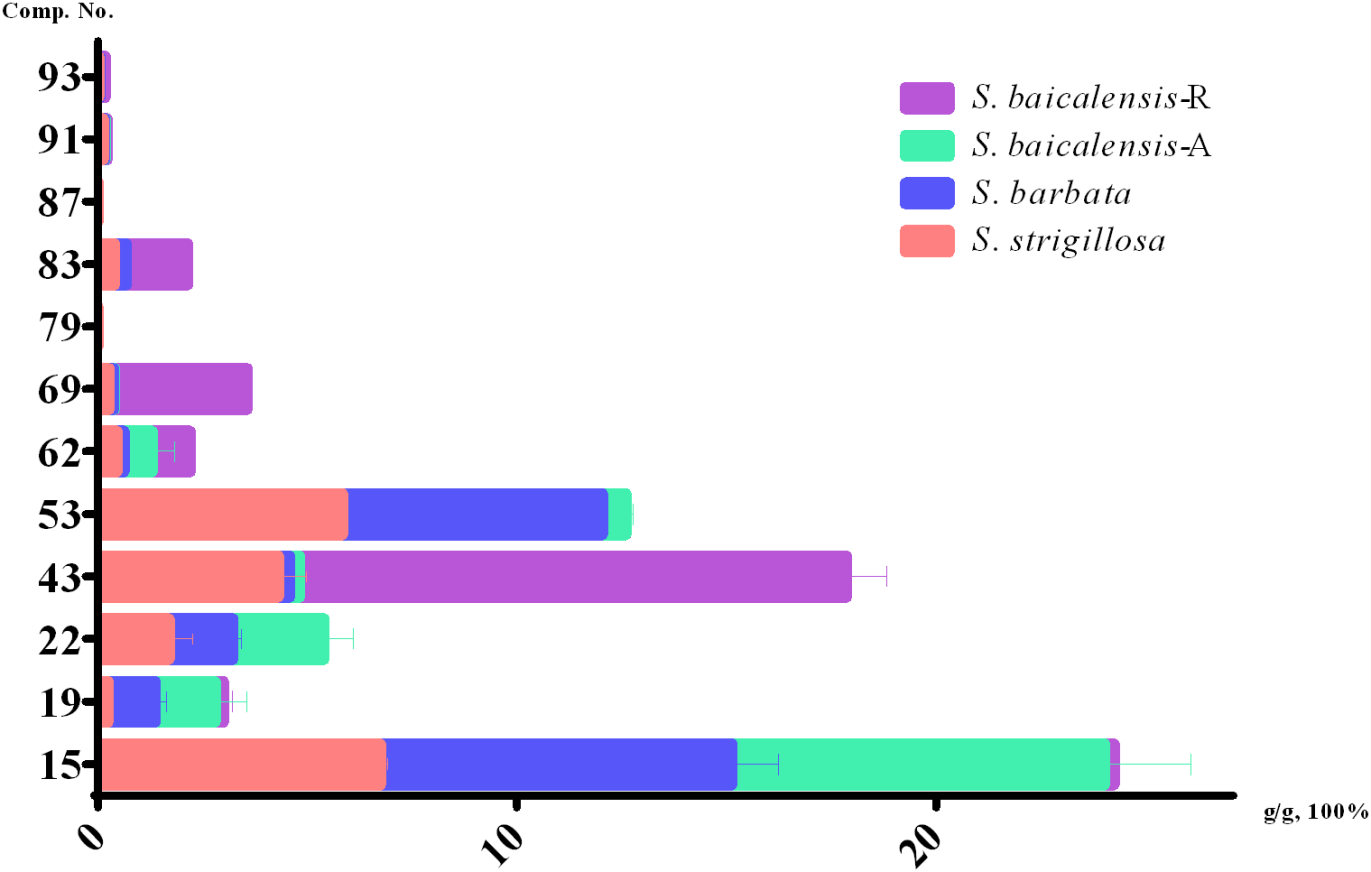
Contents of 12 compounds in *S. strigillosa, S. baicalensis* and *S. barbata* (mean ± SD, g/g 100%, n=3).

In the present study, we performed a quantitative analysis of specific phytochemicals across different *Scutellaria* species, including *S. strigillosa, S. barbata*, and two parts of *S. baicalensis*—namely, the aerial part (A) and the root (R). The concentrations of identified compounds were measured in grams per 100 grams of dry weight (g/g*100%) (**Figure 5**; **Supplementary Table 1**). The compound isocarthamidin-7-*O*-*D*-glucuronide (15) was abundant across all species, with the highest concentrations observed in *S. barbata* (8.3881 ± 1.1128%) and the aerial part of *S. baicalensis* (8.8982 ± 2.0674%). Conversely, the concentration in the roots of *S. baicalensis* was notably lower (0.2321 ± 0.0403%), as graphically represented by the shortest bar in the respective category. This suggests that the biosynthesis of this compound is significantly more active in the aerial parts of these species. The compound baicalin (43) exhibited an exceptionally high concentration in the roots of *S. baicalensis* (13.063 ± 0.9371%) and was notably abundant in *S. strigillosa* (4.3194 ± 0.6440%) and compared to other species or plant parts. This finding aligns with the traditional medicinal use of *S. baicalensis* roots, valued for their high baicalin content. Wogonoside (69) displayed a significant concentration disparity, notably higher in the roots of S. baicalensis (3.1655 ± 0.0917%) than in its aerial parts or other species. Conversely, baicalein (83) was absent in the aerial parts of *S. baicalensis* but found in its roots (1.4653 ± 0.0997%) and in the other two species, which could suggest different metabolic pathways or storage mechanisms for this metabolite within the plant. Compounds such as wogonin (87) were either absent or present only in trace amounts across all samples, which may indicate a lower biosynthetic activity or rapid metabolism. Several compounds, including scutellarin (22), isoscutellarein-8-glucuronide (53), and apigenin (79), were present in all species except in the roots of *S. baicalensis*. This absence might be attributed to differential gene expression or compound stability in the root environment. The compound baicalein (83), wogonin (87), apigenin (79), oroxylin A (93), while detected in *S. barbata* and *S. strigillosa*, and the root of *S. baicalensis*, were not observed in the aerial parts of *S. baicalensis*, which could be reflective of distinct ecological adaptations or phylogenetic divergence. Besides, oroxylin A (93) showed the highest concentration in the roots of *S. baicalensis* (0.1247 ± 0.0694%), suggesting a root-specific accumulation or synthesis.

These data highlight the complex phytochemical landscapes of *Scutellaria* species and suggest that specific metabolites are differentially synthesized and stored across plant parts and species. The distinct concentration profiles underscore the potential specialized metabolic pathways and point to each species’ unique medicinal properties. Future research should focus on elucidating the biosynthetic pathways and the ecological and pharmacological roles of these bioactive metabolites within the *Scutellaria* genus.

### 3.5 MIC determination

The MIC determination results indicate that the whole plant of *S. strigillosa* and the roots of *S. baicalensis* exhibit notable antibacterial activity, with initial inhibition rates of 45.69% and 62.54% against *Staphylococcus aureus* and 61.49% and 53.31% against *Pseudomonas aeruginosa*, respectively, and a consistent MIC of 6.00 mg/mL for both extracts and bacterial strains (**Table 2**). In contrast, *S. barbata* and the aerial parts of *S. baicalensis* showed no inhibitory effects against the tested bacterial strains (**Table 2**). These findings suggest that the roots of *S. baicalensis* and the entire *S. strigillosa* plant contain bioactive compounds responsible for their antibacterial properties, making them potential sources for developing antibacterial agents.

**Table 2:** Inhibition rates and MIC of different extracts from *Scutellaria* species against two common bacterial strains (mg/mL, $\bar{\text{x}}$ ± S, n=3).

Bacterial strains	Extracts	The initial screening inhibition rate (%)	MIC
*Staphylococcus aureus*	*S. barbata*	0.00	–
	Aerial parts of *S.baicalensis*	0.00	–
	*S.baicalensis* roots	45.69 ± 0.32	6.00 ± 0.00
	*S. strigillosa*	62.54 ± 6.38	6.00 ± 0.00
*Pseudomonas aeruginosa*	*S. barbata*	0.00	–
	Aerial parts of *S.baicalensis*	0.00	–
	*S.baicalensis* roots	61.49 ± 1.48	6.00 ± 0.00
	*S. strigillosa*	53.31 ± 1.19	6.00 ± 0.00

## 4 Discussion

In this study, the complete chloroplast genome of *S. strigillosa* was assembled for the first time, and interspecific comparison and phylogenetic analysis expanded to include *S. baicalensis* and *S. barbata* were also performed (Lee and Kim, 2019; Liu et al., 2020; Shen et al., 2022; Zhao et al., 2020). The complete chloroplast genome of *S. strigillosa* exhibited a total length of 152,533 bp. The genome size and gene content of *S. strigillosa* are not significantly different from those of most chloroplast genomes or plastomes in the genus *Scutellaria* (Shen et al., 2022). However, compared to *S. baicalensis* and *S. barbata*, *S. strigillosa*’s genome is the longest, exhibiting a total length of 152,533 bp. The comparative analysis highlighted the variability in noncoding regions, with the LSC and SSC regions showing more variability than the IR region. Highly variable regions were identified in the three *Scutellaria* species, presenting opportunities for further phylogenetic and evolutionary studies.

In the interspecific comparison, the chloroplast genomes of *S. strigillosa*, *S. baicalensis*, and *S. barbata* showed high sequence conservation, particularly within protein-coding regions. Variability was more pronounced in the LSC and SSC regions compared to the IR regions, underscoring the dynamic nature of chloroplast genome evolution. The mVISTA analysis facilitated the identification of four highly variable regions: *trnK(UUU)*-*rps16*, *trnN(GUU)* - t*rnR(ACG)*, *accD*-*psaI*, *psbE*-*petL*, which had high Pi values and could serve as potential markers for phylogenetic studies and understanding evolutionary dynamics within the genus *Scutellaria*.

*Scutellaria* is an isolated genus with unsatisfactory traditional divisions (Shen et al., 2022). The phylogenetic placement of *S. strigillosa* within the *Scutellaria* genus, based on chloroplast genome analysis, remains unexplored. This gap highlights an area for future research, where studying the chloroplast genome of *S. strigillosa* could provide insights into its evolutionary relationships within the genus. Understanding its position in the phylogenetic tree could offer valuable information about its genetic connections to other species in the *Scutellaria* genus. The phylogenetic analysis, utilizing complete chloroplast genome sequences from 28 *Scutellaria* species, classified all species into two main subclades, revealing a complex evolutionary relationship within the genus. This classification, corroborated by strong bootstrap and posterior probability values, provides a new perspective on the genetic and evolutionary relationships between species traditionally classified in different subgenera or sections within the flora of China. *S. baicalensis* and *S. barbata*, two species within the *Scutellaria* genus, are recognized in the Pharmacopoeia for their well-established medicinal properties. While *S. strigillosa* is acknowledged for its potential medicinal value, it has received comparatively less research attention. As shown in the phylogenetic tree, compared with *S. baicalensis*, *S. strigillosa* was more closely related to *S. barbata*. According to the theory of pharmacophylogenetics, *S. strigillosa* has a chemical composition and pharmacological activities that are more similar to those of *S. barbata*.

The specialized metabolite profiles across *S. strigillosa*, *S. baicalensis*, and *S. barbata* were characterized using UPLC-Q-TOF-MS analysis, highlighting the chemical diversity inherent within the genus. Notably, the chemical profile of *S. strigillosa* was closely related to *S. barbata*, reflecting their phylogenetic closeness and suggesting similar pharmacological properties. This alignment between phytochemical profiles and phylogenetic data underscores the potential of integrating genomic and metabolomic data to elucidate medicinal plants’ evolutionary trajectories and functional adaptations. Furthermore, 112 metabolites were tentatively identified. These included 3 alkaloids,1 *α-*pyrone glycoside, 30 diterpenoids, 56 flavonoids, 15 iridoid glycoside, 2 phenolics, 4 phenylethanoid glycosides, and 1 other compound. The heatmap analysis of 112 metabolites revealed distinct phytochemical variations and intricate patterns of metabolite distribution within and between the *Scutellaria* species. The differential abundance of certain metabolites across species or plant parts suggests a complex interplay of biosynthetic pathways, possibly influenced by genetic factors and ecological pressures.

The notable variance in the concentration of specific phytochemicals and the quantitative analysis of 12 assessed chemical metabolites in *S. strigillosa*, *S. baicalensis*, and *S. barbata* provide a deeper insight into the plant’s potential pharmacological applications. The results revealed significant variations in the concentration of these metabolites across different species and plant parts. Notably, isocarthamidin-7-*O*-*D*-glucuronide (15) was abundant in *S. strigillosa*, *S. barbata*, and the aerial part of *S. baicalensis*, whereas baicalin (22) showed exceptionally high concentration in the roots of *S. baicalensis*. Some compounds, like wogonin, were present in trace amounts or absent, indicating possible differences in biosynthesis or metabolism. This finding underscores their potential importance in the medicinal attributes of *S. strigillosa*. Notably, within the *Scutellaria* genus, only *S. barbata* and *S. baicalensis* are recognized in the Chinese pharmacopeia. According to these standards, *S. barbata*’s dried product should contain no less than 0.20% scutellarin, while *S. baicalensis* should comprise at least 8.00% baicalin. In our study, scutellarin (43) was detected in *S. strigillosa*, *S. barbata*, and the aerial part of *S. baicalensis*, at considerably higher concentrations, measuring 1.7067 ± 0.5478%, 1.5161 ± 0.1952%, and 2.1857 ± 0.6944% respectively. The concentration of scutellarin (43) in *S. strigillosa* and the aerial part of *S. baicalensis*, notably higher than *S. barbata*, underscores the potential medicinal value of *S. strigillosa* and the aerial part of *S. baicalensis*. The compound of baicalin (22) was detected in *S. strigillosa* 4.3194 ± 0.6440%, which is lower than the root of *S. baicalensis* but higher than others. This observation is particularly noteworthy considering the established bioactivities of these compounds, such as their antimicrobial, anti-inflammatory, antioxidant, and potential anticancer properties (Shen et al., 2021). Such findings highlight the pharmacological potential of *S. strigillosa* and suggest its possible inclusion in future revisions of pharmacopoeial standards, given the significant presence of these bioactive metabolites.

Further research has found *that S. strigillosa* demonstrates antibacterial activity against *Staphylococcus aureus* and *Pseudomonas aeruginosa*, comparable to *S.baicalensis* root extracts. Both extracts exhibit effective inhibition of these bacteria, with minimum inhibitory concentration (MIC) values of 6 mg/mL. The identification and quantification of key metabolites in *S. strigillosa*, particularly isocarthamidin-7-*O*-*D*-glucuronide (15), baicalin (22), and scutellarin (43), isoscutellarein 8-glucuronide (53), wogonoside (69), baicalein (83) revealed their presence in significant concentrations. The compounds of baicalein (83), baicalin (22), scutellarin (43), and wogonoside (69) compounds have various pharmacological activities, including antimicrobial (Jing et al., 2024; Wang et al., 2022; Zhao et al., 2022) and antitumor (Ge and Zhong, 2024; Peng et al., 2023; Ruolei et al., 2023). These findings suggest that the antibacterial properties of *S. strigillosa* may be associated with the presence of these highly abundant compounds. *S. strigillosa* could be a valuable source of antimicrobial agents. Further research is needed to elucidate the specific metabolites responsible for this activity and to understand their mechanisms of action. Additionally, exploring the potential synergistic effects with other antibiotics and assessing the safety and efficacy of these extracts *in vivo* models could pave the way for developing new therapeutic applications. Investigating their antimicrobial spectrum might also reveal potential uses against a broader range of pathogenic microorganisms.

In conclusion, our study underscores the importance of integrating genomic data, metabolomics, content analysis, and antibacterial activities to unravel the complex phytochemistry of medicinal plants of *S. strigillosa*. The insights gained from this research not only enhance our knowledge of this specific species but also highlight the potential of these plants in ecological and ornamental applications, offering new opportunities for drug discovery and the sustainable development of *S. strigillosa*.

## Data Availability

The supported genome sequence data for the findings of this study can be obtained from GenBank of NCBI (https://www.ncbi.nlm.nih.gov/) with the accession number MZ329682.

## Supplementary material

Supplementary Table 1 Contents of 12 compounds in *S. strigillosa*, *S. baicalensis*, and *S. barbata* (mean ± SD, g/g*100%, n=3).

## References

[B1] Amiryousefi A. Hyvonen J. Poczai P. (2018). IRscope: an online program to visualize the junction sites of chloroplast genomes. Bioinformatics 3417, 3030–3031. doi: 10.1093/bioinformatics/bty220 29659705

[B2] Cui Y. Chen X. Nie L. Sun W. Hu H. Lin Y.. (2019). Comparison and phylogenetic analysis of chloroplast genomes of three medicinal and edible amomum species. In.t J. Mol. Sci. 2016, 40. doi: 10.3390/ijms20164040 PMC6720276 31430862

[B3] Dai S. J. Xiao K. Zhang L. Han Q. T. (2016). New neo-clerodane diterpenoids from *Scutellaria strigillosa* with cytotoxic activities. J. Asian Natural Prod. Res. 185, 456–461. doi: 10.1080/10286020.2015.1132707 26757611

[B4] Ge H. Zhong X. (2024). Research progress on anti-tumor mechanisms of scutellarin. J. Asian Nat. Prod. Res. 55, 4608–4621. doi: 10.1080/10286020.2024.2362375 38910315

[B5] Gu R. Rybalov L. Negrin A. Morcol T. Long W. Myers A. K.. (2019). Metabolic profiling of different parts of acer truncatum from the Mongolian plateau using UPLC-Q-TOF-MS with comparative bioactivity assays. J. Agric. Food Chem. 675, 1585–1597. doi: 10.1021/acs.jafc.8b04035 30675777

[B6] Heng Z. Yuting W. Yuanquan Q. Shiyun Z. (2023). Research progress on the antibacterial resistance of heat-clearing and detoxifying drugs. Pract. Clin. J. Integrated Traditional Chin. Western Med. 2318, 125–128.

[B7] Jin J. Yu W. Yang J. Song Y. Depamphilis C. Yi T.. (2020). GetOrganelle: A fast and versatile toolkit for accurate de novo assembly of organelle genomes. Genome Biology 21, 241. doi: 10.1101/256479 32912315 PMC7488116

[B8] Jing Z. Yuhang L. Sibin X. Huadong L. Hao C. Guowen Z. (2024). Baicalein glycymicelle ophthalmic solution: Preparation, in *vitro* antimicrobial activities, and antimicrobial mechanism evaluations. Int. J. Pharm. 654, 123964. doi: 10.1016/j.ijpharm.2024.123964 38430948

[B9] Kumar S. Stecher G. Li M. Knyaz C. Tamura K. (2018). MEGA X: molecular evolutionary genetics analysis across computing platforms. Mol. Biol. Evol. 356, 1547–1549. doi: 10.1093/molbev/msy096 PMC5967553 29722887

[B10] Lee Y. Kim S. (2019). The complete chloroplast genome of Scutellaria indica var. coccinea (Lamiaceae), an endemic taxon in Korea. Mitochondrial DNA B Resour. 42, 2539–2540. doi: 10.1080/23802359.2019.1640649 PMC7706630 33365616

[B11] Li J. Wang H. Shi X. Zhao L. Lv T. Yuan Q.. (2019). Anti-proliferative and anti-migratory effects of *Scutellaria strigillosa* Hemsley extracts against vascular smooth muscle cells. J. Ethnopharmacol. 235, 155–163. doi: 10.1016/j.jep.2019.02.016 30763696

[B12] Liu X. Zuo Y. Lin L. Li W. Wang Q. Deng H. (2020). The complete chloroplast genome of Scutellaria tsinyunensis (Lamiaceae), an endemic species from China. Mitochondrial DNA B Resour. 53, 2568–2570. doi: 10.1080/23802359.2020.1781562 PMC7850432 33553625

[B13] Lukas B. Novak J. (2013). The complete chloroplast genome of Origanum vulgare L. (Lamiaceae). Gene 5282, 163–169. doi: 10.1016/j.gene.2013.07.026 23911304

[B14] Mayor C. Brudno M. Schwartz J. R. Poliakov A. Rubin E. M. Frazer K. A.. (2000). VISTA: visualizing global DNA sequence alignments of arbitrary length. Bioinformatics 1611, 1046–1047. doi: 10.1093/bioinformatics/16.11.1046 11159318

[B15] Michael T. Pascal L. Tommaso P. Ulbricht-Jones E. S. Axel F. Ralph B.. (2017). GeSeq – versatile and accurate annotation of organelle genomes. Nucleic Acids Res. 45, W1–W11. doi: 10.1093/nar/gkx391 28486635 PMC5570176

[B16] Millar B. C. Rao J. R. Moore J. E. (2021). Fighting antimicrobial resistance (AMR): Chinese herbal medicine as a source of novel antimicrobials - an update. Lett. Appl. Microbiol. 734, 400–407. doi: 10.1111/lam.13534 34219247

[B17] Nguyen L. T. Schmidt H. A. von Haeseler A. Minh B. Q. (2015). IQ-TREE: a fast and effective stochastic algorithm for estimating maximum-likelihood phylogenies. Mol. Biol. Evol. 321, 268–274. doi: 10.1093/molbev/msu300 PMC4271533 25371430

[B18] Nylander J. A. A. (2004). MrModeltest v.2. Program distributed by the author (Uppsala: Uppsala University).

[B19] Peng Y. Jingyang L. Yanqing L. Jiayi S. Yingfan H. Bo L.. (2023). Mechanistic role of Scutellaria baicalensis Georgi in breast cancer therapy. Am. J. Chin. Med. 51, 279–308. doi: 10.1142/S0192415X23500155 36655686

[B20] Quan Y. Li Z. Meng X. Li P. Wang Y. He C.. (2023). A comprehensive review of Huangqin (Scutellaria baicalensis Georgi) tea: chemical composition, functional properties and safety aspects. Beverage Plant Res. 3, 31. doi: 10.48130/BPR-2023-0031

[B21] Rozas J. Ferrer-Mata A. Sanchez-DelBarrio J. C. Guirao-Rico S. Librado P. Ramos-Onsins S. E.. (2017). DnaSP 6: DNA sequence polymorphism analysis of large data sets. Mol. Biol. Evol. 3412, 3299–3302. doi: 10.1093/molbev/msx248 29029172

[B22] Ruolei W. Chunyan W. Lianheng L. Fuwen Y. Feng H. (2023). Baicalin and baicalein in modulating tumor microenvironment for cancer treatment: A comprehensive review with future perspectives. Pharmacol. Res. 199, 107032. doi: 10.1016/j.phrs.2023.107032 38061594

[B23] Salimov R. A. Parolly G. Borsch T. (2021). Overall phylogenetic relationships of Scutellaria (Lamiaceae) shed light on the origin of the predominantly Caucasian and Irano-Turanian S. orientalis group. Willdenowia 513, 395–427. doi: 10.3372/wi.51.51307

[B24] Shen J. Li P. He C.-n. Liu H.-t. Liu Y.-z. Sun X.-b.. (2019). Simultaneous determination of 15 flavonoids from different parts of Scutellaria baicalensis and its chemometrics analysis. Chin. Herbal Medicines 111, 20–27. doi: 10.1016/j.chmed.2018.09.005

[B25] Shen J. Li P. Liu S. Liu Q. Li Y. Sun Y.. (2021). Traditional uses, ten-years research progress on phytochemistry and pharmacology, and clinical studies of the genus Scutellaria. J. Ethnopharmacol. 265, 113198. doi: 10.1016/j.jep.2020.113198 32739568

[B26] Shen J. Li P. Wang Y. Yang K. Li Y. Yao H.. (2022). Pharmacophylogenetic study of Scutellaria baicalensis and its substitute medicinal species based on the chloroplast genomics, metabolomics, and active ingredient. Front. Plant Sci. 13, 951824. doi: 10.3389/fpls.2022.951824 36061787 PMC9433114

[B27] Song J. W. Long J. Y. Xie L. Zhang L. L. Xie Q. X. Chen H. J.. (2020). Applications, phytochemistry, pharmacological effects, pharmacokinetics, toxicity of Scutellaria baicalensis Georgi. and its probably potential therapeutic effects on COVID-19: a review. Chin. Med. 151, 102. doi: 10.1186/s13020-020-00384- PMC7517065 32994803

[B28] Song X. M. Wang J. Xu Y. (2019). Study on desalination effect of 7 saline-alkali-tolerant plants. Mod. Agric. Sci. Technol. 2121, 144 + 149.

[B29] Stephan G. Pascal L. Ralph B. (2019). OrganellarGenomeDRAW (OGDRAW) version 1.3.1: expanded toolkit for the graphical visualization of organellar genomes. Nucleic Acids Res. 47, W59–W64. doi: 10.1093/nar/gkz238 30949694 PMC6602502

[B30] Sun C. Zhang M. Dong H. Liu W. Guo L. Wang X. (2020). A spatially-resolved approach to visualize the distribution and biosynthesis of flavones in Scutellaria baicalensis Georgi. J. Pharm. Biomed. Anal. 179, 113014. doi: 10.1016/j.jpba.2019.113014 31812804

[B31] Wang H. Liu Y. Cui J. Tong M. Guan W. Cao Z.. (2023). Effects of *Scutellaria strigillosa* Hemsl. extract on HepG2 cell proliferation and apoptosis through binding to aspartate β-hydroxylase. Biochem. Biophys. Res. Commun. 668, 62–69. doi: 10.1016/j.bbrc.2023.05.077 37244036

[B32] Wang X. Wei L. Wang L. Chen X. Kong X. Luan Y.. (2022). Scutellarin potentiates vancomycin against lethal pneumonia caused by methicillin-resistant Staphylococcus aureus through dual inhibition of sortase A and caseinolytic peptidase P. Biochem. Pharmacol. 199, 114982. doi: 10.1016/j.bcp.2022.114982 35247333

[B33] Wang Z. Zhu C. Liu S. He C. Chen F. Xiao P. (2019). Comprehensive metabolic profile analysis of the root bark of different species of tree peonies (Paeonia Sect. Moutan). Phytochemistry 163, 118–125. doi: 10.1016/j.phytochem.2019.04.005 31048131

[B34] Xu X. Yan S. Zhang Y. Cao L. Chen T. Yang X.. (2024). Comparison of the chemical constituents of Saposhnikoviae Radix associated with three different growth patterns and its therapeutic effect against atopic dermatitis. J. Ethnopharmacol. 333, 118417. doi: 10.1016/j.jep.2024.118417 38830452

[B35] Yan X. Liu T. Yuan X. Xu Y. Yan H. Hao G. (2019). Chloroplast Genomes and Comparative Analyses among Thirteen Taxa within Myrsinaceae s.str. Clade (Myrsinoideae, Primulaceae). Int. J. Mol. Sci. 2018, 4534. doi: 10.3390/ijms20184534 PMC6769889 31540236

[B36] Yang X. Zheng S. Wang X. Wang J. Ali shah S. B. Wang Y.. (2022). Advances in pharmacology, biosynthesis, and metabolic engineering of Scutellaria-specialized metabolites. Crit. Rev. Biotechnol., 302–318. doi: 10.1080/07388551.2022.2149386 36581326

[B37] Zhang D. Gao F. Jakovlić I. Zou H. Zhang J. Li W. X.. (2020). PhyloSuite: An integrated and scalable desktop platform for streamlined molecular sequence data management and evolutionary phylogenetics studies. Mol. Ecol. Resour. 201, 348–355. doi: 10.1111/1755-0998.13096 31599058

[B38] Zhao Q. Cui M. Y. Levsh O. Yang D. Liu J. Li J.. (2018). Two CYP82D enzymes function as flavone hydroxylases in the biosynthesis of root-specific 4’-deoxyflavones in Scutellaria baicalensis. Mol. Plant 111, 135–148. doi: 10.1016/j.molp.2017.08.009 PMC5770198 28842248

[B39] Zhao D. Du B. Xu J. Xie Q. Lu Z. Kang Y. (2022). Baicalin promotes antibacterial defenses by modulating mitochondrial function. Biochem. Biophys. Res. Commun. 621, 130–136. doi: 10.1016/j.bbrc.2022.06.084 35820283

[B40] Zhao F. Li B. Drew B. T. Chen Y. P. Wang Q. Yu W. B.. (2020). Leveraging plastomes for comparative analysis and phylogenomic inference within Scutellarioideae (Lamiaceae). PloS One 155, e0232602. doi: 10.1371/journal.pone.0232602 PMC7205251 32379799

[B41] Zhu X. Han C. Gao T. Shao H. (2016). Chemical composition, phytotoxic and antimicrobial activities of the essential oil of *Scutellaria strigillosa* Hemsley. J. Essential Oil-Bearing Plants 193, 664–670. doi: 10.1080/0972060X.2014.1000389

