# Supplementary material for: Pharmacophylogenetic insights into Scutellaria strigillosa Hemsl.: chloroplast genome and untargeted metabolomics, quantitative analysis and antibacterial analysis
Source: Front Plant Sci. 2024 Sep 25;15:1472204. doi: 10.3389/fpls.2024.1472204 (PMC11461247; doi:10.3389/fpls.2024.1472204)
Supplement: Supplementary Table 1 — Contents of 12 compounds in S. strigillosa, S. baicalensis, and S. barbata (mean ± SD, g/g*100%, n=3). [file Table1.docx]

Table S1 Contents of 12 compounds in *S. strigillosa*, *S. baicalensis*, and *S. barbata* (mean ± SD, g/g*100%, n=3)

| NO. |  | *S. barbata* | *S. strigillosa* | *S. baicalensis-*A | *S. baicalensis*-R |
| --- | --- | --- | --- | --- | --- |
| **15** | isocarthamidin-7-*O*- *D*-glucuronide | 8.3881±1.1128 | 6.7503±0.1647 | 8.8982±2.0674 | 0.2321±0.0403 |
| **19** | carthamidin-7-*O* -*D*-glucuronide | 1.1221±0.2719 | 0.2498±0.0858 | 1.4347±0.7437 | 0.1949±0.2011 |
| **22** | scutellarin | 1.5161±0.1952 | 1.7067±0.5478 | 2.1857±0.6944 | - |
| **43** | baicalin | 0.2672±0.0022 | 4.3194±0.644 | 0.237±0.0629 | 13.063±0.9371 |
| **53** | Isoscutellarein-8-*O*- glucuronide | 6.206±0.057 | 5.8439±0.0154 | 0.566±0.1645 | - |
| **62** | chrysin-7*-O*-*D*-glucuronide | 0.1653±0.0101 | 0.4571±0.074 | 0.681±0.5123 | 0.9012±0.0596 |
| **69** | wogonoside | 0.1176±0.0046 | 0.268±0.0248 | 0.0226±0.0143 | 3.1655±0.0917 |
| **79** | apigenin | 0.2097±0.0095 | 0.2338±0.0182 | 0.0212±0.0096 | - |
| **83** | Baicalein | 0.2837±0.0033 | 0.3978±0.03 | - | 1.4653±0.0997 |
| **87** | wogonin | 0.0001±0 | 0.0001±0.0001 | - | 0.0008±0.0002 |
| **91** | chrysin | 0.0309±0.0028 | 0.1381±0.0276 | 0.0078±0.0041 | 0.0513±0.0239 |
| **93** | oroxylin A | 0.0228±0 | 0.0267±0.0048 | - | 0.1247±0.0694 |
